# Hospital Digital Transformation, Nursing Data Burden, and Leadership and Governance Capacity: A Critical Narrative Review

**DOI:** 10.1155/jonm/9681970

**Published:** 2026-10-06

**Authors:** Jiang-Ying Xie, Si-Yuan Liu, Yan He

**Affiliations:** ^1^ Operating Room, Department of Anesthesiology West China Hospital Sichuan University Chengdu China scu.edu.cn; ^2^ West China School of Nursing Sichuan University Chengdu China scu.edu.cn

**Keywords:** artificial intelligence, data governance, documentation burden, electronic health records, nursing informatics, nursing leadership, nursing management, smart hospitals

## Abstract

**Aim:**

To critically synthesize how hospital digital transformation reconfigures nursing work and data burden, and to examine the distinct contributions of nurse‐leader capability and organizational governance capacity.

**Background:**

Digital systems can support documentation, surveillance, coordination, and clinical decision‐making. The same systems can add redundant entry, fragmented attention, excessive notifications, cognitive workload, and data‐quality responsibilities. Technology effects, nursing burden, and implementation leadership are often discussed separately, limiting their usefulness for nursing management.

**Design:**

Critical narrative review.

**Methods:**

A structured search of PubMed/MEDLINE and OpenAlex covered January 1, 2015, to August 21, 2026, supplemented by citation chasing and authoritative policy sources. Seventeen searches produced 8094 records before deduplication and 5735 unique records. A computer‐assisted relevance gate identified 2231 potentially relevant records, from which a rule‐based, domain‐balanced ranking generated 220 candidates. Two authors independently reviewed the candidates; 140 records underwent structured annotation, and 58 cited evidence records received detailed author‐led appraisal and claim verification. Evidence was organized across technology and work, data burden, leadership and governance, and context and equity. Interpretation was weighted by design, directness, access depth, methodological limitations, consistency, and setting. The Scale for the Assessment of Narrative Review Articles (SANRA) informed reporting quality but was not treated as a conduct standard.

**Results:**

Digital systems did not produce a uniform workload effect. Benefits were most credible when systems reduced duplicate entry, automated data transfer, improved information access, or fitted local workflow. Burden arose through documentation complexity, task switching, nonactionable alerts or alarms, verification work, and fragmented infrastructures. Individual digital competence supported adaptation but could not compensate for poor usability, inadequate staffing, weak training, or unclear accountability. Leadership capability and governance capacity operated at different levels and should not be modeled as a single mediator. Evidence from China illustrated rapid implementation and local innovation, but remained too context‐specific for national or cross‐system generalization.

**Conclusion:**

Hospital digital transformation is best evaluated as a sociotechnical reconfiguration of nursing work. Nurse managers need measures that distinguish time burden, cognitive burden, alert burden, alarm burden, and data‐quality work. Organizations need nursing representation, workflow‐centered design, continuing training, technical support, data standards, and explicit accountability. The proposed framework is an evidence‐informed organizing heuristic, not a validated causal model.

## 1. Introduction

Hospital digital transformation is not a synonym for installing an electronic health record (EHR). It is the organizational process through which digital systems, data practices, professional roles, and decision processes are reconfigured together. A smart hospital is a more specific organizational vision in which connected infrastructure, automation, sensing, and data‐driven services are integrated across clinical and administrative functions. Nursing informatics is a professional field concerned with the integration of nursing knowledge, information, and technology. The three concepts overlap, but they identify different objects: organizational change, an institutional configuration, and a professional domain, respectively. Global policy guidance similarly treats digital transformation as a health‐system and governance undertaking, not simply a technology acquisition [1–3].

Nurses occupy a central position in hospital digital work because they continuously generate, retrieve, interpret, verify, communicate, and act on clinical data. Digital systems can make information available across locations, automate transfers, support medication and monitoring processes, and reduce selected manual tasks. They can also redistribute work toward navigation, reconciliation, exception handling, and verification. Qualitative, time‐motion, and mixed‐methods studies show that observed effects vary with usability, workflow fit, interoperability, training, staffing, patient acuity, and implementation stage [4–7]. Digital technology therefore changes the composition and timing of nursing work even when total task time does not change.

The concept of nursing data burden is useful only if it is kept analytically precise. Documentation time is one dimension, not a complete definition. Burden may also arise from redundant entry, fragmented screens, repeated authentication, task switching, cognitively cumbersome navigation, low‐value fields, excessive clinical decision‐support notifications, physiologic monitor alarms, and responsibility for detecting or correcting data‐quality problems. Available measurement approaches vary substantially across studies, which makes a universal percentage of nursing shift time indefensible [6, 8–10]. Clinical decision‐support alerts and physiologic monitor alarms also require separate treatment because their signal sources, response actions, configuration decisions, and safety pathways differ.

Leadership has often been presented as the remedy for implementation problems, but the underlying construct is frequently underspecified. Individual nurse‐leader capability includes digital and artificial intelligence (AI) literacy, informatics knowledge, change leadership, communication, implementation judgment, and the ability to evaluate workflow consequences. Organizational governance capacity includes formal nursing representation, decision rights, specialist informatics roles, procurement rules, staffing and training resources, data standards, escalation routes, and accountability for performance and safety. Studies of e‐leadership, EHR training, co‐design, and organizational informatics support the relevance of both levels, but do not justify treating leadership as a causal mediator [11–14].

Prior reviews have examined documentation burden, alarm fatigue, digital competence, EHR training, AI in nursing, and technology adoption. The literature nevertheless remains difficult to translate into management decisions because constructs and outcome measures differ, empirical and normative sources are often blended, and contradictory findings are not always retained. The problem is not an absolute absence of synthesis. It is a shortage of cross‐domain synthesis that preserves distinctions among technology configuration, work mechanisms, individual capability, organizational governance, and context.

China provides a useful contextual lens because national digital‐health policy, hospital informatization, nursing workforce development, and local experimentation have progressed rapidly. Chinese studies describe participatory design, nursing‐record quality control, mobile information systems, near‐miss risks, information distortion, AI readiness, informatics competence, and technophobia [14–20]. Much of the recent evidence is cross‐sectional, single‐center, or implementation‐specific. China is therefore used throughout the review to test transferability and expose contextual conditions, not as a comprehensive Chinese‐language subgroup or a basis for national generalization.

The review asks four questions. First, how do hospital digital systems reconfigure nursing work? Second, what distinct forms of nursing data burden are reported, and how are they measured? Third, what contributions can reasonably be attributed to nurse‐leader capability and organizational governance capacity? Fourth, how do infrastructure, resources, policy, and local workflow affect transferability? The aim is to develop a management‐oriented, evidence‐informed framework while avoiding claims of exhaustive coverage, mediation, or universal effect.

## 2. Review Design and Methods

### 2.1. Design and Reporting Position

We conducted a critical narrative review because the questions span quantitative workload studies, qualitative implementation research, mixed‐methods evaluations, evidence syntheses, conceptual papers, and policy guidance. The purpose was interpretive comparison and framework development rather than exhaustive enumeration or pooled effect estimation. The Scale for the Assessment of Narrative Review Articles (SANRA) informed the clarity of the review’s importance, aims, search account, referencing, scientific reasoning, and presentation of relevant data [21]. SANRA was used as a quality and reporting checklist, not as a protocol or conduct standard. The review does not claim identity with the Preferred Reporting Items for Systematic Reviews and Meta‐Analyses (PRISMA), formal systematic‐review eligibility, or comprehensive coverage of all languages and databases.

The analytic scope was hospital and hospital‐based health systems, with attention to practicing nurses, nurse managers, nursing leaders, and nursing informatics personnel. The date emphasis was January 1, 2015, to August 21, 2026, with earlier sources retained when conceptually or methodologically important. English‐language indexed research formed the empirical core. Publicly accessible Chinese policy sources were used for contextual interpretation. Two bilingual authors reviewed the Chinese policy documents in the original Chinese, checked English title translations, and resolved interpretive differences by consensus. Table 1 defines the central constructs and boundaries.

**TABLE 1:** Operational definitions and analytic boundaries.

Construct	Operational definition in this review	Included	Excluded or kept separate
Hospital digital transformation	Organizational reconfiguration of technology, data, work, roles, and decision processes in hospital care	EHRs, nursing information systems, CDSSs, monitoring, automation, robotics, AI, and associated implementation	Technology acquisition treated as transformation without evidence of work or organizational change
Smart hospital	Hospital configuration that integrates connected infrastructure, automation, sensing, and data‐driven services	Used as an institutional descriptor when authors or policy sources define it	Used as a synonym for every digitized hospital
Nursing informatics	Professional field integrating nursing knowledge, information, and technology	Informatics roles, competence, standards, design, implementation, and governance	Generic digital literacy without nursing or hospital relevance
Nursing data burden	Effort and adverse work consequences associated with producing, locating, interpreting, reconciling, or acting on digital data beyond useful and proportionate work	Time, redundancy, navigation, fragmentation, cognitive workload, interruptions, verification, and data‐quality work	A single universal documentation‐time percentage
CDSS alert	Software‐generated message based on a rule, order, documentation event, or predictive model	Relevance, routing, actionability, overrides, and downstream consequences	Physiologic monitor alarms
Physiologic monitor alarm	Device‐generated signal based on a measured patient state, sensor condition, or technical fault	Signal quality, thresholds, sensors, response, escalation, and unit practice	CDSS alerts and override behavior
Nursing data stewardship responsibilities	Distributed nursing functions that support the clinical fitness, traceability, contextual interpretation, and appropriate use of data	Recognition, reconciliation, contextualization, escalation, and access protection	A formal occupational title, sole ownership of enterprise data quality, or responsibility for system repair
Individual leadership capability	Nurse leader’s knowledge, skills, judgment, communication, and implementation behavior	Digital and AI literacy, informatics competence, change leadership, ethics, and evaluation	Organizational resources or decision rights
Organizational governance capacity	Organizational ability to make, resource, monitor, and revise digital decisions	Representation, CNIO roles, standards, staffing, training, support, accountability, and escalation	Leadership style or personal competence alone
China contextual lens	Use of Chinese policy and empirical studies to examine context and transferability	Local illustrations compared with evidence from other settings	Comprehensive Chinese‐language synthesis, national subgroup analysis, or uniquely Chinese causal claims

Abbreviations: AI, artificial intelligence; CDSS, clinical decision‐support system; CNIO, chief nursing informatics officer.

### 2.2. Information Sources and Search Construction

PubMed/MEDLINE and OpenAlex were searched on August 21, 2026. PubMed/MEDLINE was selected for its structured coverage of clinical care, nursing informatics, health services, and hospital implementation research. OpenAlex was used as a complementary cross‐disciplinary discovery source for management, organizational, sociotechnical, implementation, and digital‐health literature that may not be indexed consistently in a biomedical database. The combination was intended to support broad purposive discovery for a critical narrative review rather than exhaustive retrieval. The Cumulative Index to Nursing and Allied Health Literature (CINAHL), Embase, Scopus, Web of Science, and specialist management databases were not searched; their omission may have excluded nursing‐management, organizational, non‐English, or discipline‐specific evidence. PubMed Central, lawful open‐access journal or repository routes, and publisher metadata supported source verification. Official sources supplied policy and professional guidance from the World Health Organization, International Council of Nurses, the National Health Commission of the People’s Republic of China, the State Council of the People’s Republic of China, United States health‐information technology authorities, and the Healthcare Information and Management Systems Society.

Three fielded PubMed search families combined hospital nursing populations with (1) digital technology and workflow or implementation; (2) digital technology and workload, documentation, alerts, alarms, cognitive burden, or data quality; and (3) digital technology and leadership, competence, governance, training, participation, or organizational support. Fourteen relevance‐oriented OpenAlex queries broadened discovery across the same domains and added low‐resource and China‐focused queries. Supporting Appendix S2 provides every executed string, search identifier, date, count, retrieval ceiling, scoring rule, quota, and warning. Supporting Dataset S4 provides the machine‐readable search log and record‐level decisions.

The 17 searches produced 8094 records before deduplication: 5294 from three complete PubMed retrievals and 2800 from fourteen OpenAlex queries. Identifier matching and normalized title‐year comparison yielded 5735 unique records. OpenAlex retrieval was limited to the 200 highest relevance‐ranked results per query even when reported counts were larger. The search is therefore structured, documented, and rerunnable within the disclosed limitations of a changing relevance‐ranked source, but it is not exhaustive. Table 2 summarizes the search and source‐selection account.

**TABLE 2:** Search execution and narrative source‐selection account.

Stage	Method and result	Quality control or limitation
Scope	Hospital nursing; January 1, 2015, to August 21, 2026; earlier seminal sources when justified	English‐language indexed evidence dominates; selected public Chinese policy sources provide context
Sources	PubMed/MEDLINE and OpenAlex; full‐text verification through PubMed Central and lawful open‐access routes; official policy and professional sources	Licensed databases were not searched and are not claimed
Search construction	Three fielded PubMed families and fourteen OpenAlex relevance queries across technology‐work, burden, leadership‐governance, and context‐equity	Exact strings, dates, identifiers, counts, and warnings retained in the search log
Deduplication	5735 unique records after identifier and normalized title‐year matching	Identifier collisions were manually resolved and logged
Retrieval	8094 records before deduplication: 5294 PubMed and 2800 OpenAlex	OpenAlex capped at 200 relevance‐ranked records per query, so coverage is not exhaustive
Computer‐assisted relevance gate	2231 records met nursing, digital‐system, and hospital or strong conceptual‐domain relevance rules	Gate and scoring rules are disclosed in Supporting Appendix S2; this stage generated candidates and did not determine final inclusion
Candidate ranking and balancing	220 candidates: technology‐work 55, data burden 65, leadership‐governance 65, context‐equity 35	Pragmatic prespecified coverage targets; exact weights, penalties, known‐item handling, and bias safeguards are disclosed
Independent human assessment and annotation	Two authors reviewed 220 candidates; 140 records retained and annotated; disagreements resolved with a third author	Record‐level decisions and annotations are included in Supporting Dataset S4
Final cited and mapped evidence	58 cited evidence records; 49 records supported or qualified 18 claims through 88 relationships	One record could inform multiple claims and one claim could have multiple sources; details appear in Supporting Table S1 and Appendix S3
Access depth	77 PMC full texts; four other open full texts; one abstract plus supporting information; 58 abstract‐only records	Non‐full‐text records were excluded from detailed, quantitative, causal, safety, and limitation claims
Appraisal	Design‐sensitive annotation of sampling, measurement, bias, temporal order, comparator, follow‐up, transferability, and source role	No numerical quality score or formal risk‐of‐bias claim
Synthesis	Deductive‐inductive coding, comparison by technology and context, contradiction register, and claim‐to‐source mapping	18 claims, 88 links: 62 direct, 23 indirect, three contradictory; all relationships were verified against full text

### 2.3. Purposeful Source Selection

Candidate construction was computer‐assisted and preceded human inclusion decisions. A rule‐based relevance gate required nursing and digital‐system signals together with hospital or strong domain relevance and retained 2231 of the 5735 unique records. Records were then ranked using title and abstract signals for nursing, hospital setting, digital technology, and four conceptual streams: technology and work, data burden, leadership and governance, and context and equity. Title matches received greater weight than abstract matches; recency contributed a small weight; and education‐only, community‐only, consumer‐facing, and technically focused records without nursing‐management relevance received penalties. Fifty‐three previously identified sources were retained as known‐item candidates for reassessment, not accepted as final evidence without review. Exact variables, weights, exclusions, and source‐code rules are reported in Supporting Appendix S2.

Before title and abstract assessment, pragmatic domain targets were set at 55 technology‐and‐work, 65 data‐burden, 65 leadership‐and‐governance, and 35 context‐and‐equity candidates. The larger allocations to burden and governance reflected their centrality to the review questions; the targets were coverage safeguards rather than estimates of evidence prevalence or quality. Two reviewers independently assessed all 220 candidates and retained 140 records for structured annotation: 35 technology and work, 42 data burden, 43 leadership and governance, and 20 context and equity. Eighty records were excluded as peripheral, redundant within a conceptual stream, protocol‐only, student‐focused without practice relevance, or technically focused without a nursing workflow or management contribution. The 140 records comprised 115 primary empirical studies, 13 evidence syntheses, and 12 conceptual or commentary sources.

Two reviewers (Jiang‐Ying Xie and Yan He) independently assessed titles and abstracts against the selection criteria. Disagreements were resolved through discussion, with Si‐Yuan Liu acting as the third reviewer when consensus could not initially be reached. Two reviewers (Jiang‐Ying Xie and Yan He) independently completed the structured critical annotations, including classification of study design, evidence role, methodological limitations, and conceptual domain. Si‐Yuan Liu performed the initial citation‐to‐claim verification, and Jiang‐Ying Xie independently checked all direct claims, quantitative statements, and disputed source interpretations against the available abstracts or full texts. Any discrepancies identified during annotation or citation verification were discussed by all three authors until consensus was reached. AI‐assisted outputs were treated as provisional: Jiang‐Ying Xie and Yan He independently reviewed them against the underlying sources, corrected inaccuracies or unsupported interpretations, and documented any unresolved concerns. All three authors reviewed and approved the final evidence set, synthesis, tables, figure, citations, and manuscript.

Selection bias from purposive allocation was addressed by retaining source provenance and scores, applying the same human eligibility criteria to known‐item and newly identified records, allowing records to contribute across conceptual streams, maintaining a contradiction register, and avoiding numerical claims about the prevalence of themes. Of the 140 annotated records, 58 were cited because they directly or contextually informed at least one final claim after appraisal for relevance, design, access depth, nonredundancy, and claim fit. The remaining 82 records informed background comparison, negative‐case and contradiction checking, and assessment of conceptual coverage but did not directly support manuscript claims. Nine additional bibliography entries were methodological or guidance anchors rather than retained evidence records. Backward and forward citation chasing was used to identify seminal, gap‐filling, or contradictory material, not to determine inclusion by citation count.

### 2.4. Critical Annotation and Appraisal

All 140 retained records received a structured annotation at the deepest level permitted by lawful access. Seventy‐seven records had PubMed Central full text, four had other open full text, one had an abstract plus Supporting Information, and 58 were abstract‐only. Each annotation recorded design, setting, technology, evidence role, key finding, direction, limitations, access depth, interpretive weight, and claim‐use restriction. The 58 records cited in the manuscript additionally underwent detailed author‐led appraisal and claim‐to‐source verification. A final audit reconciled design, role, limitations, weight, restrictions, and claim links across all files. Abstract‐only and abstract‐plus‐supplement records informed the broader evidence map but not the final claim map. Supporting Table S1 reports the verified characteristics, appraisal, interpretive weight, and restrictions for the 58 cited evidence records.

Appraisal used a common cross‐design core covering design appropriateness, population and setting relevance, measurement quality, directness, temporal and causal limitations, confounding or selection concerns, implementation‐context detail, internal limitations, consistency with other evidence, and applicability to nursing management. Design‐specific considerations were then applied: sampling, self‐report, and temporal ambiguity for cross‐sectional studies; setting, sampling, analytic transparency, and transferability for qualitative studies; observation period and task classification for time‐motion studies; comparator adequacy, confounding, follow‐up, and sustainability for intervention and pre‐post studies; and search and synthesis transparency for reviews. Interpretive weight was classified as higher, moderate, or limited/contextual. No numerical quality score or universal cutoff was used.

The appraisal identified a literature dominated by cross‐sectional surveys, qualitative studies, and single‐setting evaluations. Stronger design elements were present in time‐motion studies, longitudinal or pre‐post work, mixed‐methods evaluations, randomized or crossover studies, and evidence syntheses, but outcome definitions and contexts remained heterogeneous. Recent AI and leadership studies frequently measured attitudes, readiness, literacy, or intention rather than sustained clinical outcomes. Such evidence supports association and implementation hypotheses, not causal conclusions.

### 2.5. Synthesis and Framework Development

Evidence was coded deductively across technology configuration, nursing‐work mechanisms, data‐burden type, individual capability, organizational governance, context, and outcomes. Inductive codes captured unexpected benefits, adverse effects, null findings, workarounds, verification work, and equity concerns. Studies were compared by technology, implementation phase, setting, nursing role, evidence design, and direction of finding. Quantitative estimates were not pooled because measures, denominators, technologies, and study designs were not sufficiently comparable.

A contradiction register was maintained for five topics: EHR effects on nursing time and bedside care; alarm‐management interventions; AI efficiency versus oversight work; individual competence versus organizational capacity; and transferability of China‐specific findings. Eighteen conceptual claims were linked to 88 claim‐source relationships involving 49 of the 58 cited evidence records: 62 direct, 23 indirect, and three contradictory. The counts differ because one evidence record could inform multiple claims and each claim could be supported or qualified by multiple records. All 88 relationships were checked at full‐text locations; no claim relationship depended on abstract‐only or abstract‐plus‐supplement evidence. Supporting Appendix S3 provides the author‐verified claim‐to‐source map.

The final framework was developed after synthesis. It distinguishes technology configuration, nursing‐work mechanisms and data burden, individual leadership capability, organizational governance capacity, boundary conditions, and outcomes. It was compared conceptually with person‐task‐technology fit and sociotechnical work‐system approaches, which both direct attention away from technology as an isolated intervention [22, 23]. The framework is author‐developed, noncausal, and hypothesis‐generating. It organizes questions for implementation and evaluation; it does not assert mediation or moderation.

## 3. Digital Systems Reconfigure Nursing Work

### 3.1. Technology Categories Are Less Informative Than Work Mechanisms

Hospital digital transformation includes EHRs, nursing information systems, clinical decision‐support systems (CDSSs), connected monitors, mobile documentation, electronic handover tools, remote monitoring, robotics, automation, and AI‐assisted documentation. A technology label does not specify the work effect. Similar systems can remove a manual step in one setting and create reconciliation work in another. The more useful management question is what information moves, what task is added or removed, who becomes responsible, when exceptions occur, and how the system interacts with staffing and local routines.

EHR and electronic medical record (EMR) studies illustrate the point. Clinicians report benefits from remote information access, medication documentation, and result availability, alongside complexity, slow response, repeated logins, fragmented systems, and poor cross‐sector communication [4, 24]. National and multihospital research links favorable work environments and usability with better nurse‐reported quality, but observational work also documents frequent task switching and cognitively demanding navigation [5, 25–27]. The direction of effect is contingent on the configuration of work, not on EHR adoption alone.

Connected monitoring can likewise create both value and burden. Continuous deterioration monitoring may support surveillance outside intensive care, but adoption depends on perceived usefulness, alarm behavior, interface design, staffing, and integration with established escalation pathways [7, 28]. Remote patient monitoring studies report context‐dependent changes in nursing time and patient experience rather than automatic labor savings [29, 30]. A monitoring platform that detects change but fails to route actionable information to an accountable clinician can shift burden without improving response.

Automation and robotics demonstrate the same conditional pattern. An EHR‐connected excretion‐care system reduced selected manual work in a mixed‐methods evaluation, while broader research on nursing robots suggests that workload benefits depend on which tasks are delegated and how devices fit ward routines [31, 32]. Automation may eliminate transcription yet increase setup, troubleshooting, cleaning, exception management, or validation. Nursing managers should therefore evaluate total work‐system effects, not the duration of the automated task alone.

### 3.2. Efficiency Gains Are Real but Bounded

Several implementation studies report targeted gains. Participatory redesign of a critical‐care information system reduced documentation steps and supported bedside work in one Chinese hospital [15]. An EMR‐connected questionnaire improved selected documentation processes, and an updated electronic handover page achieved favorable adoption and usability when nurses could evaluate workflow fit [33, 34]. A randomized crossover evaluation of a spatially integrated EHR prototype reported improved usability and lower cognitive load for intensive‐care nurses [35]. Such studies show what is possible under designed conditions, but site specificity, prototype status, short follow‐up, and selected outcomes constrain generalization.

The strongest practical lesson is not that one product class is beneficial. It is that gains are more plausible when a system reduces duplicate capture, places relevant information near the decision point, preserves a coherent task sequence, and provides a clear recovery path when automation fails. Benefits are less plausible when new functionality is layered onto unchanged documentation requirements or when multiple systems remain partially interoperable. Table 3 contrasts favorable, adverse, null, and conditional findings by mechanism.

**TABLE 3:** Contrasting effects of hospital digital technologies on nursing work.

Technology or configuration	Reported benefit	Reported burden or failure mode	Critical interpretation	Management implication
EHR or EMR	Remote access, medication documentation, result availability, continuity, selected quality associations [4, 25, 26]	Complex navigation, repeated logins, parallel systems, task switching, redundant entry, mental workload [5, 6, 24, 27]	Direction varies with usability, work environment, implementation phase, and measure; adoption alone does not predict work effect	Measure workflow fit, task switching, duplicate capture, downtime, and role‐specific consequences before and after implementation
Electronic handover and structured documentation	Better information organization, selected efficiency, favorable adoption [33, 34]	New fields or rigid templates can relocate burden and omit context	Local implementation evidence is encouraging but not universally transferable	Co‐design content, test handover under realistic workload, and retire redundant fields
Connected and remote monitoring	Earlier visibility, remote surveillance, selected time or patient‐experience gains [7, 29, 30]	Alarm exposure, device setup, response coordination, technical failure, and unclear escalation	Value depends on actionable routing and response capacity, not detection alone	Define ownership, escalation, response metrics, maintenance, and balancing workload
Robotics and task automation	Reduction of selected manual or repetitive tasks [31, 32]	Setup, troubleshooting, cleaning, exception handling, and validation can create hidden work	Evaluate total work‐system change rather than task duration only	Measure work removed and added across roles, shifts, and failure states
CDSS	Can support recognition and structured decisions [22, 36]	Low‐value messages, poor timing, workflow interruption, override burden	Alert effects depend on specificity, timing, role, and governance	Use role‐sensitive routing and routine retirement or redesign of low‐value alerts
AI‐assisted documentation and prediction	Early implementations report drafting efficiency and usability benefits [37, 38]	Hallucinations, trust uncertainty, bias, verification, correction, oversight, and new alerts [39–41]	Efficiency claims are incomplete when verification and correction work are omitted	Assign verification, record provenance and corrections, monitor drift and equity, and measure net work

Abbreviations: AI, artificial intelligence; CDSS, clinical decision‐support system; EHR, electronic health record; EMR, electronic medical record.

### 3.3. AI Changes the Location of Work

AI tools extend the reconfiguration from data entry to generation, prioritization, and prediction. Current nursing evidence includes attitude and readiness surveys, qualitative studies, decision‐support prototypes, and early implementation evaluations. An AI‐assisted discharge‐summary workflow in Japan and a large language model‐supported nursing handover implementation in Taiwan reported documentation efficiencies while also examining hallucinations, safety review, and human verification [37, 38]. The contribution is promising, but the relevant workload question is whether time saved in drafting exceeds time added by checking, correcting, documenting provenance, and managing exceptions.

Nurses and nurse leaders also report uncertainty about trust, role change, legal accountability, and required competence [39, 42, 43]. A study of intended AI‐alert overrides linked self‐reported trust calibration with alert fatigue, moral distress, and team psychological safety, but its cross‐sectional design cannot establish directionality [40]. AI may reduce selected clerical tasks and simultaneously create oversight work. Calling the outcome burden reduction before measuring both sides risks repeating earlier EHR implementation errors.

AI governance must also preserve human autonomy, transparency, accountability, safety, equity, and public interest [41]. For nursing management, that requires an explicit answer to four questions: who checks an AI output, what degree of checking is expected, how correction and escalation are recorded, and how performance is monitored across populations and shifts. Without such arrangements, verification becomes invisible work assigned to bedside nurses.

### 3.4. From Technology User to Data Steward

The term data steward is sometimes used loosely. In this review, nursing data stewardship means the situated responsibility to maintain the clinical fitness, traceability, and appropriate use of data encountered in nursing work. It includes recognizing implausible values, reconciling conflicting sources, documenting context, escalating defects, and protecting appropriate access. It does not mean that an individual nurse owns the hospital’s data‐governance program, repairs system architecture, or bears sole responsibility for data quality. Nor is stewardship interchangeable with documentation, informatics competence, or clinical judgment.

Evidence on distorted electronic nursing records and nursing‐information‐system near misses shows why a distributed definition is necessary. Risks arise from system design, workflow mismatch, duplicated fields, terminology, competency, environmental pressure, and organizational conditions [18, 19, 44]. An essential clinical dataset can reduce low‐value fields, but the decision about what counts as essential requires clinical, informatics, legal, quality, and patient‐safety input [45]. Nurses contribute indispensable situated judgment, while the organization remains accountable for standards, infrastructure, staffing, and defect resolution.

## 4. Nursing Data Burden Comprises Distinct Management Problems

### 4.1. Documentation Burden is Multidimensional

Documentation burden includes the effort and adverse work consequences associated with producing, locating, interpreting, or reconciling clinical documentation beyond what is useful and proportionate for care, communication, learning, legal requirements, and accountability. The construct includes time burden, cognitive burden, fragmentation, redundancy, navigation burden, interruption, low‐value work, and perceived burden. A scoping review found substantial variation in definitions, measures, units of analysis, and professional populations [8]. Flowsheet analyses and mixed‐methods nursing studies likewise show that burden can be concentrated in particular fields, modules, or workflow transitions rather than uniformly distributed across a shift [6, 9].

Time remains important, but its interpretation depends on the denominator, observation method, role, shift, setting, implementation phase, and definition of documentation. EHR log data, direct observation, self‐report, and time‐motion studies do not measure the same thing. A mental‐health EHR analysis found variation across roles and work patterns, while time‐motion research in acute care identified task switching and documentation demands that were not captured by a single total‐time estimate [5, 10]. A universal claim that nurses spend a fixed proportion of every shift on digital documentation would erase clinically relevant variation and exceed the evidence.

Qualitative evidence adds the question of value. Nurses prioritize documentation according to patient need, legal exposure, handover requirements, and system constraints; they may postpone, compress, or work around tasks when demand exceeds capacity [46, 47]. The management problem is therefore not simply to minimize documentation. It is to reduce work that adds little clinical value while preserving information needed for care, coordination, safety, learning, and accountability [48].

### 4.2. Workflow Fragmentation and Cognitive Workload

Fragmentation occurs when a coherent clinical task is split across screens, systems, devices, authentication steps, or temporal interruptions. The nurse must remember unfinished work, reconstruct context, and reconcile inconsistent representations. Observational evidence links EHR task interruptions with mental workload, while qualitative research identifies nonintuitive design, parallel paper and digital workflows, and multiple logins as sources of friction [4, 27]. Such friction can be clinically consequential even when each additional step appears small.

Cognitive burden also depends on information architecture. A screen can contain the required data yet make comparison difficult. The randomized evaluation of a spatially integrated EHR prototype suggests that reorganizing information around nursing tasks can improve usability and cognitive load [35]. The result supports further testing of information design, but does not establish that one layout will transfer across units or specialties. Local work observation and usability testing remain necessary.

Redundancy is not always visible from record counts. The same clinical fact may be entered in multiple modules, copied into handover text, reconciled against device output, and reformatted for quality reporting. An automated intensive care unit (ICU) nursing‐record quality‐control system illustrates that improving record completeness can itself create review, reconciliation, and exception‐handling work. The management objective is not simply fewer fields, but clinically useful data produced with proportionate effort.

### 4.3. Decision‐Support Alert Burden

A clinical decision‐support alert is a software‐generated message based on a rule, order, documentation event, or predictive model. Alert burden arises when alert volume, timing, relevance, repetition, or interaction cost exceeds its decision value. Override behavior is an action taken in response to an alert; it is not a direct synonym for fatigue, inattention, or unsafe care. An override may reflect low specificity, a duplicate message, a known exception, workflow timing, or a justified clinical decision.

Nursing alert rationalization can reduce interruption when governance teams remove low‐value messages, apply role‐sensitive routing, and review alert performance. A nursing informatics‐led “discount” approach reduced interruptive nursing alert burden by systematically identifying candidates for retirement or redesign [49]. AI‐supported alerts add further concerns because model uncertainty, trust calibration, and accountability shape response [40]. Management should monitor exposure, actionability, response time, override rationale, downstream safety, and distribution across roles rather than relying on a single override rate.

### 4.4. Physiologic Monitor Alarm Burden

A physiologic monitor alarm is a device‐generated signal based on measured patient states, sensor conditions, or technical faults. Alarm burden differs from CDSS alert burden because signal generation, response urgency, bedside context, and configuration authority differ. Alarm‐management studies therefore should not be treated as direct evidence for EHR alert governance, although both illustrate the need to assess signal quality, actionability, response work, and safety outcomes separately.

Customization software and unit‐based bundles can reduce alarm rates or improve nurses’ experience in selected units [50, 51]. Sustainability depends on patient mix, electrode and sensor practice, default thresholds, staffing, escalation design, and continuing audit. A reduction in total alarms is not sufficient if clinically important signals are delayed or if work is shifted to manual surveillance. Alert and alarm governance should therefore use separate dashboards, owners, and improvement methods.

### 4.5. Data Quality, Well‐Being, and Bedside Presence

Data quality problems are often framed as failures of individual compliance. Evidence from nursing records and information‐system tasks points to a broader causal field that includes terminology, system logic, duplicated fields, competing work demands, training, staffing, and local workarounds [18, 19, 44]. When documentation systems do not match work as performed, nurses face a choice among delayed entry, approximation, duplicate work, and diversion from patient care. Accountability should follow the ability to change the contributing condition.

Poor EHR usability and insufficient implementation training have been associated with nurse dissatisfaction, well‐being concerns, and unfavorable nurse‐ or patient‐reported outcomes [12, 26]. Such associations are important, but much evidence is cross‐sectional or observational. Digital burden can plausibly compete with bedside presence and contribute to strain, yet the magnitude and direction vary. Remote monitoring and targeted automation may free time in one setting and add coordination work in another [29, 31]. The review therefore rejects deterministic claims that digital systems inevitably cause burnout or reduce bedside care.

Table 4 converts the burden taxonomy into distinct management measures and responses. The central principle is measurement before intervention. A documentation‐time problem, a navigation problem, a CDSS alert problem, a monitor‐alarm problem, and a data‐quality problem require different data and different owners.

**TABLE 4:** Taxonomy of nursing data burden and management responses.

Burden type	Observable indicators	Common contributors	Evidence strength and limitation	Proportionate management response
Documentation time burden	Time in documentation, after‐hours work, delayed entry, task duration	Mandatory fields, duplicated capture, low‐value requirements, staffing and acuity	Multiple methods show burden, but denominators and definitions vary [8, 10]	Define essential data, remove duplication, measure by role and shift, and use balancing safety measures
Navigation and fragmentation burden	Screen transitions, logins, system switching, unfinished tasks, reconciliation	Parallel systems, weak interoperability, poor information architecture	Direct observation and qualitative studies support mechanisms; site effects are large [4, 5]	Map work as done, simplify authentication, integrate views, and test real task sequences
Cognitive workload	Validated workload measures, errors, delays, perceived mental demand	Poor layout, interruptions, high information density, competing signals	Observational and experimental evidence exists, but tools and tasks differ [27, 35]	Conduct usability testing, redesign information display, protect critical task sequences
Redundancy and low‐value work	Duplicate fields, copied text, repeated reconciliation, fields rarely used	Compliance layering, unretired legacy forms, cross‐system duplication	Flowsheet and mixed‐methods evidence identifies concentrated burden [6, 9]	Create field‐retirement governance and require a named clinical purpose for retained data
CDSS alert burden	Alert exposure, actionability, response, override rationale, interruption time	Low specificity, repetition, poor timing, broad routing	Implementation studies support rationalization; override rate alone is ambiguous [40, 49]	Separate alert governance, monitor safety outcomes, and retire or redesign low‐value rules
Physiologic monitor alarm burden	Alarm rate, duration, response, false or nonactionable signals, nurse experience	Thresholds, sensor practice, device defaults, patient mix, escalation design	Reviews and unit interventions show possible reductions with heterogeneous sustainability [50–52]	Use unit‐specific signal review, customization, sensor practice, and safety balancing measures
Verification and exception work	Time checking outputs, corrections, escalations, unresolved exceptions	Automation uncertainty, AI hallucinations, poor provenance, weak recovery design	Early implementation evidence; long‐term safety and workload evidence is limited [37, 38]	Specify human review, correction, escalation, audit trail, and net‐work measurement
Data‐quality stewardship burden	Defects, conflicting values, workarounds, near misses, correction loops	Terminology, duplicated fields, workflow mismatch, unclear accountability	Qualitative and cross‐sectional evidence identifies interacting factors, not individual failure alone [18, 19, 44]	Assign enterprise ownership, improve standards and interfaces, and give frontline staff a reliable escalation path

Abbreviations: AI, artificial intelligence; CDSS, clinical decision‐support system.

## 5. Leadership Capability and Governance Capacity Operate at Different Levels

### 5.1. Individual Nurse‐Leader Capability

Nurse managers translate strategic decisions into staffing, workflow, communication, training, escalation, and daily adaptation. Digital leadership capability includes sufficient informatics and AI literacy to ask critical questions; implementation knowledge to anticipate workflow effects; communication and relational skill to surface concerns; ethical judgment; and the ability to interpret local performance data. Qualitative work with nurse managers identifies communication, change management, informatics competence, self‐efficacy, and continuing learning as relevant capabilities [11, 39].

Digital literacy and informatics competence are associated with readiness, adoption, decision‐making, work engagement, or attitudes in several settings [14, 53–56]. Most studies use cross‐sectional self‐report, and some examine intention rather than behavior. Competence scores may share method variance with outcome measures. The evidence supports investment in capability development, but not a claim that a score predicts implementation success across settings.

Leadership style studies face the same limitation. Associations between leadership style, training, EHR use, or AI literacy can identify promising mechanisms, but temporal order and organizational confounding remain unresolved [57, 58]. A digitally skilled nurse manager may work in an organization that also provides better staffing, more training, and stronger informatics support. Individual and organizational contributions must be measured separately.

### 5.2. Organizational Governance Capacity

Governance capacity is the organization’s ability to make, resource, monitor, and revise digital‐system decisions. It includes nursing representation in procurement and design; formal roles such as the chief nursing informatics officer (CNIO); decision rights for documentation, alerts, alarms, and data standards; technical and analytic support; implementation resources; accountability; and mechanisms for reporting and correcting harm. The CNIO is a senior nursing informatics role that connects nursing practice, executive strategy, information technology, and data governance. Professional guidance describes the role as broader than system implementation alone [59].

Co‐design and participatory design are concrete governance mechanisms. Case studies of critical‐care systems and EHR nursing summaries show that nurse participation can reveal information needs, sequence problems, and usability barriers [13, 15]. Supporting evidence is mainly qualitative, case‐based, or implementation‐specific. Co‐design should therefore be treated as a plausible mechanism that requires evaluation, not a guarantee of better outcomes.

Training is another organizational responsibility. Research on inpatient EHR training and implementation suggests that training must be role‐specific, timed to workflow, reinforced after go‐live, and supported by accessible troubleshooting [12, 60]. One‐time instruction cannot compensate for system changes, staff turnover, or evolving workarounds. Protected learning time, super‐user networks, postimplementation rounds, and feedback loops are governance investments rather than optional individual remediation.

Data standards and accountability are equally important. Standardized nursing terminology may support interoperability and reuse, but knowledge, attitudes, practice, system design, and institutional support all affect adoption [44]. Organizations need explicit ownership for data definitions, field retirement, quality rules, model inputs, and correction pathways. Assigning data stewardship to bedside nurses without the authority or resources to change system defects is responsibility without control.

### 5.3. Why Capability Cannot Substitute for Capacity

The distinction between capability and capacity prevents two management errors. The first is blaming nurses or managers for poor adoption when the system is unusable, understaffed, or weakly supported. The second is creating governance structures without developing the people who must recognize and act on local consequences. Individual competence and organizational capacity are complementary but noninterchangeable.

Mixed‐methods and qualitative evidence repeatedly identify workflow fit, technical support, staffing, training, and organizational climate alongside user competence [6, 7, 11]. A leader may identify a harmful alert but lack authority to change its rule. A governance committee may authorize redesign but fail to understand bedside sequence. Effective implementation requires a connection between situated nursing knowledge and formal decision rights.

Table 5 separates capability, capacity, evidence limits, and practical actions. The distinction also guides future measurement. Individual measures should assess knowledge, skill, judgment, behavior, and learning. Organizational measures should assess representation, resources, response time, decision rights, data standards, training infrastructure, and the reliability of improvement loops. Outcomes should be assessed independently rather than embedded within a readiness score.

**TABLE 5:** Individual leadership capability, organizational governance capacity, and practical actions.

Level	Component	What it enables	Evidence and limitation	Practical action for nursing management
Individual capability	Digital, informatics, and AI literacy	Critical questioning, informed use, recognition of limitations	Associations with readiness and attitudes are mostly cross‐sectional and self‐reported [14, 53, 56]	Assess role‐specific learning needs and provide protected, continuing education
Individual capability	Implementation and change leadership	Communication, local adaptation, learning, and concern escalation	Qualitative nurse‐manager evidence supports relevance; outcome evaluations are scarce [11, 39]	Train managers to map workflow, interpret implementation data, and run feedback cycles
Individual capability	Ethical and evaluative judgment	Recognition of safety, equity, accountability, and verification needs	Normative guidance is strong; empirical intervention evidence is early [3, 41]	Include ethics, equity, data provenance, and failure recovery in manager development
Organizational capacity	Nursing representation and decision rights	Translation of bedside knowledge into procurement, design, configuration, and review	Co‐design evidence is promising but mainly case‐based [13, 15]	Consider formal voting or documented consultation rights for practicing nurses and nurse managers where governance structures permit, alongside escalation routes and local evaluation
Organizational capacity	CNIO and nursing informatics infrastructure	Connection among practice, executive strategy, IT, analytics, and data governance	Professional guidance supports role definition; comparative outcome evidence is limited [59]	Define authority, resources, unit links, and response‐time expectations for informatics roles
Organizational capacity	Continuing training and technical support	Safe adaptation across go‐live, system change, and staff turnover	Reviews and surveys support continuing support; delivery models vary [12, 60]	Budget protected training time, post‐go‐live rounds, super‐users, and accessible troubleshooting
Organizational capacity	Documentation, alert, alarm, AI, and distributed data‐stewardship governance	Mechanism‐specific standards, monitoring, correction, and accountability	Evidence supports problem separation; effectiveness varies by intervention and setting	Create separate owners and dashboards with balancing safety and workload outcomes
Boundary conditions	Staffing, infrastructure, interoperability, patient population, policy, and resources	Feasibility and transferability	Low‐resource and China‐focused studies show strong context dependence [61–64]	Pilot locally, stratify measures, assess total implementation cost, and revise before scale‐up

Abbreviations: AI, artificial intelligence; CNIO, chief nursing informatics officer; IT, information technology.

## 6. Context Determines Transferability

### 6.1. Infrastructure, Resources, and Organizational Setting

Digital interventions depend on infrastructure reliability, device availability, interoperability, technical support, staffing, and local care pathways. Evaluation of monitoring technology in low‐resource hospitals in Malawi highlighted the need to adapt technology to local constraints and user priorities [61]. Research in Ethiopian hospitals found that EMR use and satisfaction were shaped by system and implementation conditions rather than by availability alone [62]. A technically effective tool may be infeasible when connectivity, maintenance, replacement parts, or staffing are unreliable.

Resource constraints do not justify lower safety or usability standards. They change the sequence and scale of implementation. A lower‐resource hospital may gain more from reliable identity management, essential datasets, and stable handover than from adding an advanced predictive model. Investment guidance emphasizes alignment with health‐program priorities, total implementation cost, governance, and workforce capability [2]. Nurse managers should be included when deciding which problem deserves scarce implementation capacity.

Digital inequality also exists within well‐resourced systems. Units differ in staffing, patient acuity, device density, and access to informatics support. Night‐shift, agency, temporary, and rotating staff may receive less training. AI tools may perform differently across populations and settings. Equity assessment should include who receives benefit, who performs added work, whose data are represented, and who can challenge an output.

### 6.2. China as a Contextual Lens

China’s national health‐informatization plans position digital infrastructure, data sharing, governance, and health‐service modernization as strategic priorities [64, 65]. Nursing development policy has also emphasized service capacity and workforce development. Policy direction creates momentum, but national intent does not establish local implementation outcomes. Hospital level, region, specialty, workforce, vendor environment, and management capacity influence how a policy becomes nursing work.

Chinese empirical studies illustrate both innovation and risk. Participatory critical‐care system design reported improved documentation workflow in one hospital [15]. An ICU electronic nursing‐record quality‐control system reported gains in selected quality‐control processes [16]. A randomized study of a 5G‐based nursing information system reported lower work‐stress scores in its study setting [17]. At the same time, qualitative research on information distortion and multicenter survey research on near misses identified interacting system, workflow, competency, and organizational factors [18, 19].

Recent Chinese studies of AI readiness, literacy, technophobia, and standardized nursing terminology expand the picture but are largely cross‐sectional and often region‐ or hospital‐specific [20, 44, 63, 66]. Such studies can identify distributions and associations, but cannot establish that leadership produces literacy or that readiness causes safe adoption. A head‐nurse digital leadership study that uses mediation analysis may estimate relationships within its model, but it does not validate a universal causal chain for digital transformation [67].

The Chinese evidence converges with research elsewhere on the importance of workflow fit, training, user participation, data standards, and organizational support. It also highlights how rapid scale‐up can make interoperability, unequal capability, and accountability more visible. The main inference is contextual: policy acceleration increases the importance of local work‐system evaluation. The review does not claim that the identified patterns are unique to China or representative of every Chinese hospital.

## 7. An Evidence‐Informed Nursing Digital Transformation Work‐System Framework

Figure 1 presents a noncausal work‐system heuristic with six components. Technology configuration describes functions, interfaces, automation, integration, and the implementation stage. Nursing‐work mechanisms describe how tasks, information, attention, and responsibility are redistributed. Data burden is represented within the work‐mechanism layer and separated into documentation, cognitive, fragmentation, alert, alarm, verification, and data‐quality work. Individual leadership capability and organizational governance capacity are shown as distinct implementation resources. Boundary conditions include staffing, infrastructure, policy, professional roles, patient population, and resource setting. Outcomes include work quality, patient safety, nurse well‐being, equity, data fitness, and intended operational or clinical benefits.

**FIGURE 1:**
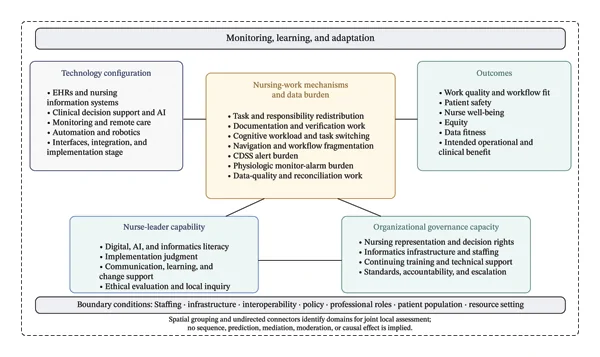
Evidence‐informed nursing digital transformation work‐system framework. The author‐developed figure is a noncausal, hypothesis‐generating organizing heuristic and has not been empirically validated. Spatial grouping and undirected connectors identify domains for joint local assessment; they do not represent temporal sequence, prediction, mediation, moderation, or causal effects. Technology configuration, nursing‐work mechanisms and data burden, nurse‐leader capability, organizational governance capacity, boundary conditions, and outcomes remain analytically distinct. Nurse‐leader capability and governance capacity are proposed implementation resources for prospective evaluation, not demonstrated modifiers of technology effects. Monitoring, learning, and adaptation are cross‐cutting activities. AI, artificial intelligence; CDSS, clinical decision‐support system; EHR, electronic health record.

The framework does not encode a sequence or tested effect. Spatial grouping and undirected relationships identify domains that should be assessed together during design, implementation, and evaluation. Technology configuration, work mechanisms and data burden, implementation resources, boundary conditions, and outcomes remain analytically distinct. Nurse‐leader capability and organizational governance capacity are propositions for local assessment and prospective testing; neither has been demonstrated to mediate, moderate, or otherwise modify technology effects. Monitoring, learning, and adaptation are represented as a cross‐cutting activity rather than a directional causal loop.

The framework’s contribution is comparative and nursing‐management‐specific. Sociotechnical work‐system frameworks emphasize interactions among people, tasks, technologies, organization, and environment but do not necessarily separate nursing data‐burden mechanisms or nursing decision authority. Person‐task‐technology fit frameworks identify alignment problems but give less attention to governance infrastructure, staffing, accountability, and balancing outcomes. Implementation‐science frameworks identify determinants and implementation outcomes but do not usually distinguish nurse‐leader capability from organizational governance capacity. Nursing‐informatics competency frameworks clarify individual knowledge and skills but may not capture enterprise decision rights, infrastructure, and work redistribution. The proposed heuristic brings these concerns into one assessment structure without claiming that its component concepts are new or causally linked.

The heuristic generates propositions rather than conclusions. Workflow fit may be associated with less low‐value burden when staffing and implementation support are adequate. Nursing representation could support earlier identification and correction of workflow defects when representatives have meaningful decision rights. Continuing training may be more useful than one‐time training as systems and staff change. Alert and alarm interventions could be evaluated with safety outcomes and work redistribution, not only volume. AI documentation tools may be evaluated on the network after verification and correction. Each proposition requires prospective testing with multilevel designs.

## 8. Discussion

### 8.1. Principal Interpretation

The central management question is not whether a technology was adopted or users are ready, but how implementation redistributes nursing tasks, attention, responsibility, and risk; whether the resulting work has clinical value; and whether unintended effects can be detected and corrected.

Evidence was heterogeneous across EHRs, monitoring, automation, and AI. Mixed findings identified implementation contingencies rather than noise: benefit was more plausible when systems removed low‐value work without creating equivalent hidden work.

The burden synthesis also shows why a single headline percentage or override rate is misleading. Documentation, navigation, task switching, CDSS alerts, physiologic alarms, verification, and data‐quality work have different mechanisms and owners. A hospital can reduce documentation time while increasing cognitive or verification burden. It can lower alarm counts while making escalation more complex. Evaluation needs a small portfolio of mechanism‐specific measures plus balancing outcomes.

Nurse managers can recognize workflow problems, support learning, communicate risk, and use local data, but enterprise interoperability, procurement, resources, and data standards require organizational authority. The framework therefore keeps individual capability and governance capacity separate and avoids recasting organizational failure as a readiness deficit.

### 8.2. Implications for Nursing Management

The evidence supports five proportionate actions. First, establish a baseline before implementation. Map current work, document task variation by role and shift, and measure burden and safety signals relevant to the intended change. Second, give practicing nurses and nurse managers formal roles in design, procurement, configuration, and postimplementation review. Participation should include decision rights and a documented response to identified concerns.

Third, separate governance pathways. Documentation governance should address essential data, duplication, terminology, and field retirement. CDSS alert governance should address relevance, routing, frequency, actionability, overrides, and downstream outcomes. Monitor‐alarm governance should address signal quality, thresholds, sensors, escalation, and unit practice. AI governance should add model scope, data provenance, performance drift, human verification, correction, and equity.

Fourth, treat training and support as continuing infrastructure. Competence needs will differ by role and implementation phase. Nurse managers require enough technical and analytic literacy to question a system and enough organizational support to act on the answer. Superusers and CNIO structures can connect frontline knowledge with enterprise decisions, but roles, escalation paths, and protected time must be explicit.

Fifth, evaluate network and balancing outcomes. Measures should include intended benefit, time, cognitive workload, interruptions, data‐quality defects, workarounds, well‐being, patient safety, and equity where feasible. New digital work should be assigned, resourced, and visible. A saved minute in one department should not be counted as an organizational gain if it creates 2 min of nursing reconciliation elsewhere.

The actions are evidence‐informed, not mandates proven effective in all settings. Hospitals should pilot, measure, and revise them. Table 5 identifies the strength and limitation of the supporting evidence so that recommendations remain proportionate.

### 8.3. Ethical and Equity Considerations

Digital governance is also ethical governance. Automation can obscure how a recommendation was produced, while intensive monitoring can expand surveillance of patients and staff. Documentation simplification may omit information valued by patients or specific clinical groups. AI errors can be distributed unevenly across populations, and verification work can be shifted to staff with the least authority. World Health Organization guidance places human autonomy, transparency, accountability, inclusiveness, safety, and public interest at the center of AI governance [41].

Nursing management has a distinct contribution because nurses observe downstream effects across the full care cycle. Ethical review should include how consent and patient expectations are handled, who can contest an output, how errors are reported, whether staff surveillance is proportionate, and whether workload or benefit is distributed fairly. Low‐resource settings and under‐resourced units should not become sites for poorly supported technology simply because the need is high.

Where digital systems affect communication, consent, surveillance, or access to care, patient and family representatives may also contribute to design and governance review alongside practicing nurses.

Cybersecurity, automation bias, deskilling, privacy, and secondary data use also require defined ownership, monitoring, and escalation. Governance should specify who can access data, who evaluates failures or inequitable effects, who may suspend unsafe automation, and how staff and patients can contest consequential outputs.

### 8.4. Research Priorities

Future research should move from readiness and attitude measures toward prospective, multilevel evaluation. Studies should measure technology configuration, work mechanisms, individual capability, governance capacity, boundary conditions, and outcomes separately. Interrupted time‐series, stepped‐wedge, cluster, longitudinal mixed‐methods, and natural‐experiment designs could improve causal inference while retaining context.

Burden measurement requires harmonization without collapsing distinct constructs. A core measurement set could include documentation time and after‐hours work, task switching, cognitive workload, duplicate entry, alert exposure and actionability, alarm exposure and response, verification time, data‐quality defects, workarounds, and balancing safety outcomes. Measures should be stratified by role, shift, unit, patient acuity, and implementation stage when feasible.

Leadership research should test behaviors and organizational mechanisms, not only self‐rated competence. Studies could evaluate whether nursing representation with formal decision rights changes defect resolution, whether CNIO‐supported governance improves response time, or whether continuing training changes safe system use. Comparative work across regions and resource settings should examine transferability rather than treating country as a simple covariate.

AI research should report the full labor pathway from data input to output review, correction, communication, and accountability. Efficiency estimates that omit verification work are incomplete. Safety evaluation should include hallucinations, automation bias, alert burden, downtime, model drift, equity, and recovery when outputs are wrong.

### 8.5. Strengths and Limitations

The review’s strengths are its explicit conceptual boundaries, documented search account, balanced evidence streams, separation of empirical and normative sources, design‐sensitive appraisal, author‐verified claim‐to‐source mapping, and deliberate retention of contradictory findings. The framework was developed after synthesis and is explicitly author‐developed, evidence‐informed, noncausal, hypothesis‐generating, and not empirically validated.

Several limitations remain. Narrative source selection was purposive and cannot establish exhaustive coverage. Only PubMed/MEDLINE and OpenAlex were searched directly; omission of CINAHL, Embase, Scopus, Web of Science, and specialist management databases may have excluded relevant nursing‐management, organizational, non‐English, or discipline‐specific evidence. OpenAlex used a changing relevance‐ranked ceiling of 200 records per query. The computational relevance rules, domain quotas, and known‐item prioritization could favor the concepts used to organize the review despite independent human reassessment and contradiction searching. The evidence set is English‐language dominant, and the China stream is not a comprehensive Chinese‐language review. Citation chasing can reinforce well‐connected literature despite gap‐filling rules.

The empirical literature is heterogeneous and dominated by cross‐sectional, self‐report, qualitative, and single‐setting studies. Measures of burden, readiness, usability, and outcomes differ. Recent AI and digital‐leadership studies have limited follow‐up and may be vulnerable to publication, novelty, and common‐method biases. Abstract‐only and abstract‐plus‐supplement records informed the broader evidence mapping but were excluded from the final claim‐to‐source map. No formal meta‐analysis or pooled effect estimate was attempted, and the proposed framework has not been empirically validated.

## 9. Conclusion

Hospital digital transformation can improve selected nursing tasks and create new forms of work. The balance depends on technology configuration, workflow fit, staffing, training, support, decision rights, and local context. Documentation burden, cognitive burden, CDSS alert burden, physiologic alarm burden, verification work, and data‐quality work should be measured separately.

The evidence supports nursing participation, continuing support, mechanism‐specific governance, and evaluation of unintended consequences. It does not support a universal documentation‐time estimate, a deterministic link from digitalization to burnout, or leadership readiness as a causal mediator. Nurse‐leader capability and organizational governance capacity are distinct and complementary. The author‐developed framework offers a practical agenda for local inquiry and prospective testing, not a validated prediction model.

### 9.1. Implications for Nursing Management

Nurse managers should use the framework to identify what work a digital system removes, adds, redistributes, or hides. Organizations should pair individual capability development with formal nursing representation, clear decision rights, continuing training, technical support, and separate governance for documentation, decision‐support alerts, monitor alarms, data quality, and AI. Local pilots should measure intended benefit, burden, safety, well‐being, and equity before scale‐up.

## Author Contributions

Jiang‐Ying Xie, Si‐Yuan Liu, and Yan He: conceptualization, Jiang‐Ying Xie and Yan He: methodology, Jiang‐Ying Xie, Si‐Yuan Liu, and Yan He: investigation, Jiang‐Ying Xie and Si‐Yuan Liu: data curation, Jiang‐Ying Xie, Si‐Yuan Liu, and Yan He: formal analysis, Jiang‐Ying Xie and Yan He: writing–original draft, Jiang‐Ying Xie, Si‐Yuan Liu, and Yan He: writing–review and editing, Si‐Yuan Liu, and Yan He: visualization, Jiang‐Ying Xie: supervision, and Jiang‐Ying Xie and Yan He: project administration.

## Funding

No external funding was received.

## Conflicts of Interest

The authors declare no conflicts of interest.

## Supporting Information

Additional supporting information can be found online in the Supporting Information section.

## Supporting information


**Supporting Information 1** The following supporting information accompanies this article: Supporting Table S1: Characteristics, critical appraisal, interpretive weighting, and claim‐use restrictions for the 58 cited evidence records.

**Supporting Information 2** Supporting Appendix S2: Complete search strategy and source‐selection record, including the executed PubMed/MEDLINE and OpenAlex searches, retrieval counts, deduplication procedures, relevance‐ranking rules, and selection process.

**Supporting Information 3** Supporting Appendix S3: Claim‐to‐source map linking 18 conceptual claims to 88 verified source relationships involving 49 cited evidence records.

**Supporting Information 4** Supporting Dataset S4: Machine‐readable workbook containing the search log, 220‐record candidate pool, 140‐record annotation set, 58‐record evidence matrix, 88 claim–source relationships, and source‐selection rules.

## Data Availability

No primary participant data were generated. Materials supporting the review are provided as Supporting Table S1 (the 58‐record evidence and appraisal matrix), Supporting Appendix S2 (the complete search and source‐selection record), Supporting Appendix S3 (the claim‐to‐source map), and Supporting Dataset S4 (machine‐readable search logs, candidate records and dispositions, evidence annotations, appraisal data, and claim–source relationships). Copyrighted full texts are not redistributed.
