# Supplementary material for: Hospital Digital Transformation, Nursing Data Burden, and Leadership and Governance Capacity: A Critical Narrative Review
Source: J Nurs Manag. 2026 Oct 6;2026:9681970. doi: 10.1155/jonm/9681970 (PMC13639933; doi:10.1155/jonm/9681970)
Supplement: Supplementary file 1 [file JONM-2026-9681970-s001.docx]

**Supplementary Table S1. Characteristics, appraisal, and claim-use restrictions for cited evidence records**

Scope: This table summarizes 58 evidence records cited in the manuscript. Methodological and guidance references are excluded. A final record-by-record audit reconciled study design, evidence role, methodological limitations, interpretive weight, and claim-use restrictions across the manuscript and supplementary files.

| **No.** | **Citation / PMID** | **Country or context** | **Design / source role** | **Technology or topic** | **Access depth** | **Appraisal / weight** | **Use in synthesis** |
| --- | --- | --- | --- | --- | --- | --- | --- |
| 1 | Clej et al. (2026) PMID 42512579 | Romania | cross-sectional survey; primary empirical | data burden; clinical decision support; alarms alerts or monitoring; artificial intelligence | pmc full text | Flags: convenience sampling; cross sectional no temporal inference; self report or common method risk; single site. Weight: moderate with design specific caveats. | Orientation: burden or risk signal. Restriction: Supports descriptive distributions and associations; does not establish temporal order or causal effects. |
| 2 | Tong et al. (2026) PMID 41952446 | China | cross-sectional study; primary empirical | leadership governance; artificial intelligence | pmc full text | Flags: convenience sampling; cross sectional no temporal inference; self report or common method risk. Weight: moderate with design specific caveats. | Orientation: capability governance or implementation condition. Restriction: Supports descriptive distributions and associations; does not establish temporal order or causal effects. |
| 3 | Sun et al. (2026) PMID 41992642 | China | multicenter cross-sectional study; primary empirical | context equity; digital health or informatics | pmc full text | Flags: cross sectional no temporal inference; self report or common method risk. Weight: moderate with design specific caveats. | Orientation: context transferability or equity condition. Restriction: Supports descriptive distributions and associations; does not establish temporal order or causal effects. |
| 4 | Taylor-Vaughan et al. (2026) PMID 42486461 | not reported in title or abstract | randomized crossover trial; primary empirical | technology work; EHR or EMR | pmc full text | Flags: single center; simulated or prototype task; crossover carryover possible; short term outcomes. Weight: higher for narrative synthesis. | Orientation: technology work reconfiguration. Restriction: Supports short-term within-study comparative effects; transferability to routine clinical implementation remains uncertain. |
| 5 | Alruwaili et al. (2026) PMID 42140235 | Saudi Arabia | cross-sectional descriptive correlational study; primary empirical | leadership governance; clinical decision support; digital health or informatics | pmc full text | Flags: cross sectional no temporal inference; convenience sampling; self report; three hospitals. Weight: moderate with design specific caveats. | Orientation: capability or implementation condition. Restriction: Supports associations between informatics competency and perceived decision-making; not causal effects. |
| 6 | Feng et al. (2026) PMID 41609141 | China | single-center cross-sectional survey; primary empirical | context equity; digital health or informatics | open pdf full text | Flags: cross sectional no temporal inference; self report or common method risk; single site. Weight: moderate with design specific caveats. | Orientation: context transferability or equity condition. Restriction: Supports descriptive distributions and associations; does not establish temporal order or causal effects. |
| 7 | Chen et al. (2026) PMID 41819121 | Taiwan | multihospital before-after implementation study; primary empirical | data burden; EHR or EMR; artificial intelligence; digital health or informatics | pmc full text | Flags: nonrandomized before after; log data; no concurrent control; early implementation period. Weight: moderate with design specific caveats. | Orientation: mixed or conditional burden effect. Restriction: Supports observed documentation-time changes after implementation; not causal effectiveness or safety conclusions. |
| 8 | Nakano et al. (2026) PMID 42326256 | Japan | single-center before-after implementation study; primary empirical | technology work; EHR or EMR; artificial intelligence | pmc full text | Flags: nonrandomized before after comparison; small samples; single site; short term evaluation; non nursing population. Weight: limited or contextual. | Orientation: AI documentation efficiency and verification work. Restriction: Indirect contextual support for AI documentation efficiency and verification work; not nursing-specific outcome evidence. |
| 9 | Wu et al. (2026) PMID 42345517 | China | time-lagged observational study; primary empirical | context equity; artificial intelligence | pmc full text | Flags: time lagged observational design; self report; common method risk; no causal mediation or moderation inference. Weight: moderate with design specific caveats. | Orientation: context transferability or equity condition. Restriction: Supports time-lagged associations; mediation and moderation estimates do not validate causal mechanisms. |
| 10 | Pan et al. (2026) PMID 41873534 | not reported in title or abstract | convergent mixed-methods study; primary empirical | technology work; EHR or EMR; alarms alerts or monitoring | pmc full text | Flags: multicountry convenience sample; cross sectional quantitative component; small interview sample; context heterogeneity. Weight: higher for narrative synthesis. | Orientation: technology work reconfiguration. Restriction: Supports integrated implementation and usability findings within the studied settings; transferability remains context dependent. |
| 11 | Mugoya et al. (2026) PMID 42057876 | not reported in title or abstract | phenomenological qualitative study; primary empirical | data burden; EHR or EMR | pmc full text | Flags: context specific transferability. Weight: moderate with design specific caveats. | Orientation: mixed or conditional burden effect. Restriction: Supports context-specific experiences and themes; does not estimate prevalence, comparative effects, or causality. |
| 12 | Kontodimopoulos et al. (2026) PMID 42042656 | Greece | cross-sectional survey; primary empirical | technology work; artificial intelligence; digital health or informatics | pmc full text | Flags: cross sectional no temporal inference; self report or common method risk. Weight: moderate with design specific caveats. | Orientation: technology work reconfiguration. Restriction: Supports descriptive distributions and associations; does not establish temporal order or causal effects. |
| 13 | Li et al. (2026) PMID 42271407 | China | cross-sectional latent-profile analysis; primary empirical | context equity; artificial intelligence | pmc full text | Flags: convenience sampling; cross sectional no temporal inference; self report or common method risk; single site. Weight: moderate with design specific caveats. | Orientation: context transferability or equity condition. Restriction: Supports descriptive distributions and associations; does not establish temporal order or causal effects. |
| 14 | Yu et al. (2026) PMID 41867482 | not reported in title or abstract | cross-sectional study; primary empirical | context equity; artificial intelligence | pmc full text | Flags: convenience sampling; cross sectional no temporal inference; self report or common method risk. Weight: moderate with design specific caveats. | Orientation: context transferability or equity condition. Restriction: Supports descriptive distributions and associations; does not establish temporal order or causal effects. |
| 15 | Eriksson et al. (2025) PMID 41436270 | Sweden | qualitative study; primary empirical | data burden; EHR or EMR | pmc full text | Flags: context specific transferability; convenience sampling; single site. Weight: moderate with design specific caveats. | Orientation: technology work reconfiguration. Restriction: Supports context-specific experiences and themes; does not estimate prevalence, comparative effects, or causality. |
| 16 | Kemp et al. (2025) PMID 41349583 | Canada | retrospective observational EHR-usage study; primary empirical | data burden; EHR or EMR | pmc full text | Flags: single site; retrospective audit log proxies; observational no causal inference; setting specific workflow. Weight: moderate with design specific caveats. | Orientation: mixed or conditional burden effect. Restriction: Supports setting-specific EHR-usage and documentation-time patterns; not causal effects. |
| 17 | Berkhout et al. (2025) PMID 40996085 | Netherlands | qualitative study; primary empirical | leadership governance; clinical decision support; alarms alerts or monitoring | pmc full text | Flags: purposive sample; semistructured interviews; context specific transferability; framework guided interpretation. Weight: moderate with design specific caveats. | Orientation: capability governance or implementation condition. Restriction: Supports qualitative CDSS fit, adoption, and workflow themes; does not estimate prevalence or effects. |
| 18 | Ruan et al. (2025) PMID 39923066 | not reported in title or abstract | randomized controlled trial; primary empirical | data burden; digital health or informatics | pmc full text | Flags: convenience sampling; single site; single center; self reported work stress; implementation context. Weight: higher for narrative synthesis. | Orientation: burden reduction signal. Restriction: Supports the study's comparative effect estimate; generalizability remains limited by the single implementation context. |
| 19 | Göktepe et al. (2025) PMID 40528654 | not reported in title or abstract | qualitative descriptive study; primary empirical | leadership governance; artificial intelligence | pmc full text | Flags: purposive sample; small sample; self report; context specific transferability. Weight: moderate with design specific caveats. | Orientation: capability governance or implementation condition. Restriction: Supports qualitative nurse-manager perspectives on AI-related work and governance; not effect estimates. |
| 20 | Ayed et al. (2025) PMID 40598327 | not reported in title or abstract | cross-sectional survey; primary empirical | technology work; artificial intelligence | pmc full text | Flags: convenience sampling; cross sectional no temporal inference; self report or common method risk. Weight: moderate with design specific caveats. | Orientation: technology work reconfiguration. Restriction: Supports descriptive distributions and associations; does not establish temporal order or causal effects. |
| 21 | Song et al. (2025) PMID 40503520 | China | two-phase scoping review and cross-sectional survey; multiphase evidence synthesis and primary empirical | data burden; robotics | pmc full text | Flags: scoping review depends on search and included studies; cross sectional patient preference survey; hypothetical workload reduction. Weight: moderate with design specific caveats. | Orientation: mixed or conditional burden effect. Restriction: Supports mapped robot functions and patient preferences; does not demonstrate realized nursing-workload reduction. |
| 22 | Liu et al. (2025) PMID 41272773 | China | cross-sectional correlational study; primary empirical | leadership governance; artificial intelligence; digital health or informatics | pmc full text | Flags: cross sectional no temporal inference; self report; mediation no causal inference; regional sample. Weight: moderate with design specific caveats. | Orientation: capability governance or implementation condition. Restriction: Supports associations among AI literacy, anxiety, and attitudes; mediation estimates are not causal pathways. |
| 23 | Jedwab et al. (2025) PMID 41149684 | Australia | pre-post mixed-methods study; primary empirical | data burden; EHR or EMR; digital health or informatics | pmc full text | Flags: nonrandomized pre post; single organization; self report; implementation context may limit transferability. Weight: moderate with design specific caveats. | Orientation: mixed or conditional burden effect. Restriction: Supports adoption and usability findings within one implementation; does not establish causal effects. |
| 24 | Abou et al. (2025) PMID 40726545 | Saudi Arabia | qualitative descriptive study; primary empirical | leadership governance; digital health or informatics | pmc full text | Flags: purposive sample; single hospital; self report; context specific transferability. Weight: moderate with design specific caveats. | Orientation: capability governance or implementation condition. Restriction: Supports nurse-manager perspectives on digital transformation and informatics capability; not outcome effects. |
| 25 | Du et al. (2025) PMID 40696341 | China | multicenter cross-sectional study; primary empirical | data burden; digital health or informatics | pmc full text | Flags: convenience sampling; cross sectional no temporal inference; self report or common method risk. Weight: moderate with design specific caveats. | Orientation: mixed or conditional burden effect. Restriction: Supports descriptive distributions and associations; does not establish temporal order or causal effects. |
| 26 | Wang et al. (2025) PMID 40202777 | China | qualitative study; primary empirical | data burden; clinical decision support | pmc full text | Flags: context specific transferability; single site. Weight: moderate with design specific caveats. | Orientation: technology work reconfiguration. Restriction: Supports context-specific experiences and themes; does not estimate prevalence, comparative effects, or causality. |
| 27 | Park et al. (2025) PMID 40068168 | United States | exploratory co-design mixed-methods case study; primary empirical | leadership governance; EHR or EMR | pmc full text | Flags: single site; purposive sample; exploratory co design; simulated or prototype summary. Weight: moderate with design specific caveats. | Orientation: capability governance or implementation condition. Restriction: Supports co-design preferences and information-fit themes; not implemented clinical or workload outcomes. |
| 28 | Chung et al. (2025) PMID 41430256 | Korea | cross-sectional study; primary empirical | leadership governance; digital health or informatics | open pdf full text | Flags: convenience sampling; cross sectional no temporal inference; self report or common method risk. Weight: moderate with design specific caveats. | Orientation: capability governance or implementation condition. Restriction: Supports descriptive distributions and associations; does not establish temporal order or causal effects. |
| 29 | Alnomasy et al. (2025) PMID 40631348 | Saudi Arabia | descriptive correlational cross-sectional study; primary empirical | leadership governance; artificial intelligence | pmc full text | Flags: cross sectional no temporal inference; self report; convenience sample; context specific transferability. Weight: moderate with design specific caveats. | Orientation: capability governance or implementation condition. Restriction: Supports nurse-leader perceptions and preparedness associations; not causal or longitudinal effects. |
| 30 | Guo et al. (2024) PMID 38443955 | China | cross-sectional survey; primary empirical | leadership governance; digital health or informatics | pmc full text | Flags: cross sectional no temporal inference; self report or common method risk. Weight: moderate with design specific caveats. | Orientation: capability governance or implementation condition. Restriction: Supports descriptive distributions and associations; does not establish temporal order or causal effects. |
| 31 | Lloyd et al. (2024) PMID 36866778 | Australia | qualitative analysis of free-text survey responses; primary empirical | technology work; EHR or EMR; digital health or informatics | pmc full text | Flags: free text subset; self selection of comments; mixed clinician sample; context specific transferability. Weight: moderate with design specific caveats. | Orientation: technology work reconfiguration. Restriction: Supports qualitative usability and workflow themes; does not estimate prevalence or comparative effects. |
| 32 | Nguyen et al. (2024) PMID 39225789 | not reported in title or abstract | scoping review; evidence synthesis | context equity; EHR or EMR | pmc full text | Flags: review conclusions depend on search and included study quality; scoping review; heterogeneous training programs; no effect synthesis. Weight: moderate with design specific caveats. | Orientation: context transferability or equity condition. Restriction: Supports the mapped range of evidence and implementation approaches; not pooled effectiveness estimates. |
| 33 | L et al. (2024) PMID 38966455 | India | mixed-methods observational evaluation; primary empirical | data burden; digital system unspecified | pmc full text | Flags: nonrandomized ward comparison; single hospital; time motion and self report; residual confounding. Weight: moderate with design specific caveats. | Orientation: mixed or conditional burden effect. Restriction: Supports context-specific time-motion and experience findings; not a causal estimate of remote-monitoring effects. |
| 34 | Rayan et al. (2024) PMID 38440198 | not reported in title or abstract | qualitative study; primary empirical | data burden; alarms alerts or monitoring; digital health or informatics | pmc full text | Flags: context specific transferability; convenience sampling. Weight: moderate with design specific caveats. | Orientation: mixed or conditional burden effect. Restriction: Supports context-specific experiences and themes; does not estimate prevalence, comparative effects, or causality. |
| 35 | Chirambo et al. (2024) PMID 39711002 | not reported in title or abstract | mixed-methods usability evaluation; primary empirical | context equity; alarms alerts or monitoring | pmc full text | Flags: small sample; mixed methods usability evaluation; low resource context; no clinical effectiveness test. Weight: limited or contextual. | Orientation: context transferability or equity condition. Restriction: Supports usability and co-design considerations in a low-resource setting; not clinical-effectiveness claims. |
| 36 | Cho et al. (2024) PMID 39259920 | not reported in title or abstract | mixed-methods study; primary empirical | data burden; EHR or EMR; clinical decision support | pmc full text | Flags: single hospital; small interview sample; log data and self report; context specific transferability. Weight: higher for narrative synthesis. | Orientation: mixed or conditional burden effect. Restriction: Supports integrated implementation and usability findings within the studied settings; transferability remains context dependent. |
| 37 | Zhang et al. (2024) PMID 39026330 | China | prospective controlled trial; primary empirical | data burden; EHR or EMR | pmc full text | Flags: nonrandomized controlled design; single site; short follow up; context specific transferability. Weight: moderate with design specific caveats. | Orientation: burden reduction signal. Restriction: Supports context-specific differences after implementation; does not establish effects beyond the study setting. |
| 38 | Wathne et al. (2024) PMID 39166216 | Norway | qualitative study; primary empirical | technology work; digital system unspecified | pmc full text | Flags: context specific transferability; convenience sampling. Weight: moderate with design specific caveats. | Orientation: technology work reconfiguration. Restriction: Supports context-specific experiences and themes; does not estimate prevalence, comparative effects, or causality. |
| 39 | Thompson et al. (2024) PMID 38876466 | not reported in title or abstract | quality-improvement before-after evaluation; primary empirical | data burden; clinical decision support; alarms alerts or monitoring | pmc full text | Flags: quality improvement design; before after comparison; single health system; concurrent changes possible. Weight: moderate with design specific caveats. | Orientation: mixed or conditional burden effect. Restriction: Supports local alert-rationalization outcomes; not a controlled causal effect estimate. |
| 40 | Shan et al. (2023) PMID 36890555 | not reported in title or abstract | prospective observational study; primary empirical | data burden; EHR or EMR | pmc full text | Flags: single site; observational no causal inference; context specific workload measurement. Weight: moderate with design specific caveats. | Orientation: EHR interruption and cognitive-workload mechanism. Restriction: Supports observed associations among interruptions, workload, and task performance; does not establish causal pathways. |
| 41 | Moon et al. (2023) PMID 37902820 | not reported in title or abstract | qualitative focus-group study; primary empirical | data burden; EHR or EMR; robotics; digital health or informatics | pmc full text | Flags: purposive and snowball sampling; single organization; anticipated benefits not observed outcomes. Weight: moderate with design specific caveats. | Orientation: mixed or conditional burden effect. Restriction: Supports nurses' anticipated uses and burden-reduction expectations; not measured workload reduction. |
| 42 | Kodama et al. (2023) PMID 37634203 | not reported in title or abstract | multimethod observational evaluation; primary empirical | data burden; EHR or EMR | pmc full text | Flags: nonrandomized use group comparison; single site; patient self selection; context specific workflow. Weight: moderate with design specific caveats. | Orientation: mixed or conditional burden effect. Restriction: Supports context-specific documentation-time and usability findings; not causal or universally transferable effects. |
| 43 | Hani et al. (2022) PMID 35699627 | Jordan | cross-sectional survey; primary empirical | leadership governance; EHR or EMR | pmc full text | Flags: convenience sampling; cross sectional no temporal inference; self report or common method risk. Weight: moderate with design specific caveats. | Orientation: capability governance or implementation condition. Restriction: Supports descriptive distributions and associations; does not establish temporal order or causal effects. |
| 44 | Jung et al. (2021) PMID 33830061 | United States | phenomenological qualitative study; primary empirical | technology work; EHR or EMR; clinical decision support; alarms alerts or monitoring | pmc full text | Flags: purposive sample; four behavioral hospitals; context specific transferability. Weight: moderate with design specific caveats. | Orientation: technology work reconfiguration. Restriction: Supports qualitative implementation-barrier and facilitator themes; does not estimate prevalence or effects. |
| 45 | Moy et al. (2021) PMID 33434273 | not reported in title or abstract | scoping review; evidence synthesis | context equity; EHR or EMR | pmc full text | Flags: scoping review; heterogeneous definitions and measures; conclusions depend on search and included studies. Weight: higher for narrative synthesis. | Orientation: context transferability or equity condition. Restriction: Supports the range and inconsistency of documentation-burden measures; not pooled burden estimates. |
| 46 | Kennedy et al. (2021) PMID 35308926 | not reported in title or abstract | targeted literature review; evidence synthesis | technology work; EHR or EMR; clinical decision support; alarms alerts or monitoring | pmc full text | Flags: targeted pubmed review; non systematic selection; heterogeneous implementation studies. Weight: limited or contextual. | Orientation: technology work reconfiguration. Restriction: Supports contextual claims about CDSS implementation and alert fatigue; not pooled or comprehensive effect estimates. |
| 47 | Kutney-Lee et al. (2021) PMID 33797506 | not reported in title or abstract | cross-sectional linked-data analysis; primary empirical | technology work; EHR or EMR | pmc full text | Flags: cross sectional no temporal inference; linked administrative and survey data; residual confounding. Weight: higher for narrative synthesis. | Orientation: technology work reconfiguration. Restriction: Supports adjusted associations between EHR usability and nurse or patient outcomes; does not establish causality. |
| 48 | Heponiemi et al. (2021) PMID 34941546 | not reported in title or abstract | cross-sectional survey; primary empirical | leadership governance; EHR or EMR; digital health or informatics | pmc full text | Flags: cross sectional no temporal inference; self report or common method risk. Weight: moderate with design specific caveats. | Orientation: capability governance or implementation condition. Restriction: Supports descriptive distributions and associations; does not establish temporal order or causal effects. |
| 49 | Moy et al. (2020) PMID 33936464 | not reported in title or abstract | time-motion observational study; primary empirical | data burden; EHR or EMR | pmc full text | Flags: implementation context may limit transferability. Weight: higher for narrative synthesis. | Orientation: burden or risk signal. Restriction: Supports observed workflow and time-allocation patterns; does not establish causal effects beyond the observed settings. |
| 50 | Lewandowska et al. (2020) PMID 33202907 | not reported in title or abstract | systematic review; evidence synthesis | context equity; alarms alerts or monitoring | pmc full text | Flags: review conclusions depend on search and included study quality; systematic review; heterogeneous studies and measures; no pooled effect estimate. Weight: higher for narrative synthesis. | Orientation: context transferability or equity condition. Restriction: Supports qualitative synthesis of alarm-fatigue effects within heterogeneous studies; not a pooled causal estimate. |
| 51 | Howarth et al. (2020) PMID 33148237 | not reported in title or abstract | multimethod development and usability evaluation; primary empirical | technology work; EHR or EMR; clinical decision support | pmc full text | Flags: development stage evaluation; small usability sample; single health system; no clinical effect estimate. Weight: moderate with design specific caveats. | Orientation: technology work reconfiguration. Restriction: Supports user-centered CDSS development, acceptability, and usability claims; not clinical-effectiveness claims. |
| 52 | Sutton et al. (2020) PMID 32643778 | not reported in title or abstract | multiorganizational consensus and implementation evaluation; primary empirical | data burden; EHR or EMR | pmc full text | Flags: consensus based selection; early adopter evaluation; no concurrent control; organization specific implementation. Weight: moderate with design specific caveats. | Orientation: mixed or conditional burden effect. Restriction: Supports consensus-based documentation reduction and early implementation findings; not a controlled effectiveness estimate. |
| 53 | Lewis et al. (2019) PMID 30946125 | not reported in title or abstract | quality-improvement pre-post evaluation; primary empirical | data burden; alarms alerts or monitoring | abstract plus supplement | Flags: abstract and supplement only; quality improvement design; single unit; before after comparison; concurrent changes possible. Weight: limited or contextual. | Orientation: mixed or conditional burden effect. Restriction: Broad contextual support for unit-level alarm-bundle implementation only; not detailed effectiveness or causal claims. |
| 54 | Kutney-Lee et al. (2019) PMID 30786302 | not reported in title or abstract | secondary cross-sectional analysis; primary empirical | technology work; EHR or EMR | pmc full text | Flags: cross sectional no temporal inference; self report or common method risk. Weight: moderate with design specific caveats. | Orientation: technology work reconfiguration. Restriction: Supports descriptive distributions and associations; does not establish temporal order or causal effects. |
| 55 | Ruppel et al. (2018) PMID 30335824 | not reported in title or abstract | pre-post intervention study; primary empirical | data burden; EHR or EMR; alarms alerts or monitoring | pmc full text | Flags: convenience sampling; implementation context may limit transferability; nonrandomized pre post; single unit; concurrent changes possible; self reported nurse experience. Weight: higher for narrative synthesis. | Orientation: mixed or conditional burden effect. Restriction: Supports local before-after alarm and nurse-experience findings; concurrent changes and lack of randomization limit causal inference. |
| 56 | Collins et al. (2018) PMID 30815074 | not reported in title or abstract | retrospective descriptive EHR-data analysis; primary empirical | data burden; EHR or EMR | pmc full text | Flags: descriptive log data; limited sites; documentation entries are burden proxies; no outcome comparator. Weight: moderate with design specific caveats. | Orientation: mixed or conditional burden effect. Restriction: Supports descriptive estimates of flowsheet-entry burden; not universal shift-time percentages or causal effects. |
| 57 | Qin et al. (2017) PMID 29212480 | China | descriptive case study with pre-post evaluation; primary empirical | data burden; digital health or informatics | pmc full text | Flags: single site case study; nonrandomized pre post; concurrent changes possible; context specific transferability. Weight: moderate with design specific caveats. | Orientation: mixed or conditional burden effect. Restriction: Supports participatory-design and local before-after implementation findings; not a causal or generalizable effect estimate. |
| 58 | Tilahun et al. (2015) PMID 26007237 | Ethiopia | cross-sectional evaluation study; primary empirical | context equity; EHR or EMR; digital health or informatics | pmc full text | Flags: cross sectional no temporal inference; self report or common method risk. Weight: moderate with design specific caveats. | Orientation: context transferability or equity condition. Restriction: Supports descriptive distributions and associations; does not establish temporal order or causal effects. |

Abbreviations: AI, artificial intelligence; CDSS, clinical decision-support system; EHR, electronic health record; EMR, electronic medical record; PMC, PubMed Central.
