# Supplementary material for: Hospital Digital Transformation, Nursing Data Burden, and Leadership and Governance Capacity: A Critical Narrative Review
Source: J Nurs Manag. 2026 Oct 6;2026:9681970. doi: 10.1155/jonm/9681970 (PMC13639933; doi:10.1155/jonm/9681970)
Supplement: Supplementary file 2 [file JONM-2026-9681970-s002.docx]

**Supplementary Appendix S2. Search strategy and source-selection record**

**Date window:** 1 January 2015 to 21 August 2026.

**Sources searched:** PubMed/MEDLINE and OpenAlex.

**S2.1 Retrieval accounting**

| **Source or stage** | **Searches** | **Records** | **Status** |
| --- | --- | --- | --- |
| PubMed/MEDLINE | 3 | 5,294 | Complete retrieval |
| OpenAlex | 14 | 2,800 | Top 200 relevance-ranked records per query |
| Combined before deduplication | 17 | 8,094 | All retrieved records |
| Unique pool | n/a | 5,735 | After identifier and normalized title-year deduplication |
| Automated relevance gate | n/a | 2,231 | Potentially relevant records before ranked candidate construction |
| Balanced candidate pool | n/a | 220 | Rule-based ranking with pragmatic prespecified domain targets; final inclusion remained human |
| Retained evidence pool | n/a | 140 | 140 retained after independent assessment by two authors; 80 excluded |
| Final cited evidence set | n/a | 58 | 58 records linked to 18 claims through 88 author-verified relationships |

**S2.2 Execution and deduplication procedures**

**PubMed/MEDLINE:** All records returned by the three searches were retrieved through the PubMed application programming interface. The search date and publication-date limits were embedded in the executed strings and preserved in the machine-readable log.

**OpenAlex:** Each targeted query was sorted by relevance and limited to the 200 highest-relevance records. Every query exceeded the retrieval ceiling. The resulting search is therefore non-exhaustive, which is consistent with the critical narrative review design and is disclosed in the manuscript.

**Deduplication:** Records were normalized and matched first by stable identifiers, including DOI and PMID, and then by normalized title and publication year. Identifier and title-year matches were merged while retaining source provenance and query membership. The combined pool of 8,094 retrieved records produced 5,735 unique records.

Candidate construction: A rule-based gate identified 2,231 potentially relevant records from the 5,735 unique records. Records were ranked using nursing, hospital, digital-system, and domain-relevance signals. A purposive quota retained 220 candidates across technology and nursing work, data burden, leadership and governance, and context and equity. Candidate status did not constitute final inclusion; two authors independently assessed the 220 candidates.

Warnings: OpenAlex reported result counts exceeded the 200-record ceiling for all fourteen queries. The search is non-exhaustive and may underrepresent sources indexed preferentially in CINAHL, Embase, Scopus, Web of Science, specialist management databases, non-English databases, or lower-ranked OpenAlex results. Two identifier-collision warnings were manually resolved before evidence use.

S2.3 Operational candidate-ranking and source-selection rules

Automated relevance gate. Records were eligible for ranking when they contained nursing and digital-system signals together with either a hospital-setting signal or strong relevance to one of the four conceptual streams. Known-item candidates from the earlier evidence set were allowed through the gate for reassessment.

Core scoring. Nursing signals received four points per title match and two per abstract match; hospital-setting signals received three points per title match and one per abstract match; digital-technology signals received three points per title match and one per abstract match. The core bridge score was capped at six nursing, five hospital, and six digital points.

Domain scoring. For technology-work, data-burden, leadership-governance, and context-equity terms, title matches received four points and abstract matches one point. Publications from 2020 onward received two recency points; publications from 2015-2019 received one. Each out-of-scope education, community, consumer, or weakly relevant topic signal incurred a three-point penalty.

Known-item handling. Fifty-three previously identified sources received a 40-point contribution to their mapped domain and an 80-point overall prioritization bonus during candidate construction so that they were not lost during automated ranking. They were retained as known-item candidates, not accepted as final evidence. The same human eligibility, appraisal, and claim-verification requirements were subsequently applied to them.

Candidate targets. Before title and abstract assessment, pragmatic coverage targets were set at 55 technology-work, 65 data-burden, 65 leadership-governance, and 35 context-equity records. The larger data-burden and leadership-governance allocations reflected the central review questions. Targets were not estimates of evidence prevalence, strength, or quality.

Human assessment and bias safeguards. Two authors independently assessed all 220 candidates; disagreements were resolved by discussion and, when needed, a third author. Bias safeguards included retaining record-level scores and provenance, applying the same criteria to known-item and newly retrieved sources, permitting cross-domain contribution, maintaining a contradiction register, and not treating domain counts as prevalence estimates.

Transition to 140 and 58 records. Human assessment retained 140 records: 35 technology-work, 42 data-burden, 43 leadership-governance, and 20 context-equity. The 58 cited evidence records were selected after appraisal for claim relevance, design, access depth, nonredundancy, and contribution to the final synthesis. The other 82 records informed background comparison, negative-case and contradiction checks, and conceptual-coverage assessment but did not directly support manuscript claims.

Relationship counts. Forty-nine of the 58 cited evidence records contributed to 18 conceptual claims through 88 relationships because one record could inform multiple claims and each claim could draw on multiple records. The 88 relationships comprised 62 direct, 23 indirect, and three contradictory links. All 88 relationships were verified against full-text locations.

S2.4 Exact PubMed/MEDLINE searches

**F1 | search_pubmed_f1_20260821_narrative**

**Execution:** Reported and retrieved records: 2,793. Retrieval limit: 10,000. Status: complete. Search window: 1 January 2015 to 21 August 2026.

("Nurses"[Mesh] OR "Nursing Staff, Hospital"[Mesh] OR "Nurse Administrators"[Mesh] OR "Nursing Supervisory"[Mesh] OR "Nursing Informatics"[Mesh] OR nurse*[tiab] OR "nursing staff"[tiab] OR "registered nurse*"[tiab] OR "clinical nurse*"[tiab] OR "nurse manager*"[tiab] OR "nurse leader*"[tiab] OR "head nurse*"[tiab] OR "chief nursing officer*"[tiab] OR "nursing administrator*"[tiab] OR "nursing informatic*"[tiab]) AND
("Hospitals"[Mesh] OR "Hospital Units"[Mesh] OR hospital*[tiab] OR inpatient*[tiab] OR "acute care"[tiab] OR ward*[tiab] OR "clinical unit*"[tiab] OR "tertiary hospital*"[tiab] OR "teaching hospital*"[tiab]) AND
("Electronic Health Records"[Mesh] OR "Decision Support Systems, Clinical"[Mesh] OR "Artificial Intelligence"[Mesh] OR "Internet of Things"[Mesh] OR "Medical Informatics Applications"[Mesh] OR "Automation"[Mesh] OR "Telemedicine"[Mesh] OR "Monitoring, Physiologic"[Mesh] OR "electronic health record*"[tiab] OR "electronic medical record*"[tiab] OR EHR[tiab] OR EMR[tiab] OR "clinical information system*"[tiab] OR "hospital information system*"[tiab] OR "nursing information system*"[tiab] OR "clinical decision support"[tiab] OR CDSS[tiab] OR "artificial intelligence"[tiab] OR "machine learning"[tiab] OR "internet of things"[tiab] OR IoT[tiab] OR "smart hospital*"[tiab] OR "smart ward*"[tiab] OR "digital transformation"[tiab] OR "digital health"[tiab] OR "ambient clinical documentation"[tiab] OR "ambient documentation"[tiab] OR "speech recognition"[tiab] OR "robotic process automation"[tiab] OR "workflow automation"[tiab] OR "barcode medication administration"[tiab] OR "closed-loop medication"[tiab] OR "remote patient monitoring"[tiab] OR telehealth[tiab]) AND
("Documentation"[Mesh] OR "User-Computer Interface"[Mesh] OR "Diffusion of Innovation"[Mesh] OR "Organizational Innovation"[Mesh] OR "Implementation Science"[Mesh] OR workflow*[tiab] OR "work process*"[tiab] OR "nursing documentation"[tiab] OR "clinical documentation"[tiab] OR usability[tiab] OR "user experience"[tiab] OR "technology acceptance"[tiab] OR "technology adoption"[tiab] OR implement*[tiab] OR integration[tiab] OR "work system*"[tiab] OR "work-as-done"[tiab] OR "time and motion"[tiab] OR "task shifting"[tiab] OR workaround*[tiab]) AND
("2015-01-01"[Date - Publication] : "2026-08-21"[Date - Publication])

**F2 | search_pubmed_f2_20260821_narrative**

**Execution:** Reported and retrieved records: 676. Retrieval limit: 10,000. Status: complete. Search window: 1 January 2015 to 21 August 2026.

("Nurses"[Mesh] OR "Nursing Staff, Hospital"[Mesh] OR "Nurse Administrators"[Mesh] OR "Nursing Supervisory"[Mesh] OR "Nursing Informatics"[Mesh] OR nurse*[tiab] OR "nursing staff"[tiab] OR "registered nurse*"[tiab] OR "clinical nurse*"[tiab] OR "nurse manager*"[tiab] OR "nurse leader*"[tiab] OR "head nurse*"[tiab] OR "chief nursing officer*"[tiab] OR "nursing administrator*"[tiab] OR "nursing informatic*"[tiab]) AND
("Hospitals"[Mesh] OR "Hospital Units"[Mesh] OR hospital*[tiab] OR inpatient*[tiab] OR "acute care"[tiab] OR ward*[tiab] OR "clinical unit*"[tiab] OR "tertiary hospital*"[tiab] OR "teaching hospital*"[tiab]) AND
("Electronic Health Records"[Mesh] OR "Decision Support Systems, Clinical"[Mesh] OR "Artificial Intelligence"[Mesh] OR "Internet of Things"[Mesh] OR "Medical Informatics Applications"[Mesh] OR "Automation"[Mesh] OR "Telemedicine"[Mesh] OR "Monitoring, Physiologic"[Mesh] OR "electronic health record*"[tiab] OR "electronic medical record*"[tiab] OR EHR[tiab] OR EMR[tiab] OR "clinical information system*"[tiab] OR "hospital information system*"[tiab] OR "nursing information system*"[tiab] OR "clinical decision support"[tiab] OR CDSS[tiab] OR "artificial intelligence"[tiab] OR "machine learning"[tiab] OR "internet of things"[tiab] OR IoT[tiab] OR "smart hospital*"[tiab] OR "smart ward*"[tiab] OR "digital transformation"[tiab] OR "digital health"[tiab] OR "ambient clinical documentation"[tiab] OR "ambient documentation"[tiab] OR "speech recognition"[tiab] OR "robotic process automation"[tiab] OR "workflow automation"[tiab] OR "barcode medication administration"[tiab] OR "closed-loop medication"[tiab] OR "remote patient monitoring"[tiab] OR telehealth[tiab]) AND
("Workload"[Mesh] OR "Burnout, Professional"[Mesh] OR "Alert Fatigue, Health Personnel"[Mesh] OR workload*[tiab] OR "work burden"[tiab] OR "documentation burden"[tiab] OR "documentation time"[tiab] OR "clerical burden"[tiab] OR "time burden"[tiab] OR "cognitive workload"[tiab] OR "cognitive burden"[tiab] OR "cognitive load"[tiab] OR "information overload"[tiab] OR "alert fatigue"[tiab] OR "alarm fatigue"[tiab] OR "alert burden"[tiab] OR "alarm burden"[tiab] OR "notification overload"[tiab] OR "nonactionable alert*"[tiab] OR technostress[tiab] OR "digital stress"[tiab] OR "workflow fragmentation"[tiab] OR "workflow interruption*"[tiab] OR "work interruption*"[tiab] OR workaround*[tiab] OR burnout[tiab] OR "emotional exhaustion"[tiab] OR "data quality"[tiab] OR "data integrity"[tiab] OR "data stewardship"[tiab]) AND
("2015-01-01"[Date - Publication] : "2026-08-21"[Date - Publication])

**F3 | search_pubmed_f3_20260821_narrative**

**Execution:** Reported and retrieved records: 1,825. Retrieval limit: 10,000. Status: complete. Search window: 1 January 2015 to 21 August 2026.

("Nurses"[Mesh] OR "Nursing Staff, Hospital"[Mesh] OR "Nurse Administrators"[Mesh] OR "Nursing Supervisory"[Mesh] OR "Nursing Informatics"[Mesh] OR nurse*[tiab] OR "nursing staff"[tiab] OR "registered nurse*"[tiab] OR "clinical nurse*"[tiab] OR "nurse manager*"[tiab] OR "nurse leader*"[tiab] OR "head nurse*"[tiab] OR "chief nursing officer*"[tiab] OR "nursing administrator*"[tiab] OR "nursing informatic*"[tiab]) AND
("Hospitals"[Mesh] OR "Hospital Units"[Mesh] OR hospital*[tiab] OR inpatient*[tiab] OR "acute care"[tiab] OR ward*[tiab] OR "clinical unit*"[tiab] OR "tertiary hospital*"[tiab] OR "teaching hospital*"[tiab]) AND
("Electronic Health Records"[Mesh] OR "Decision Support Systems, Clinical"[Mesh] OR "Artificial Intelligence"[Mesh] OR "Internet of Things"[Mesh] OR "Medical Informatics Applications"[Mesh] OR "Automation"[Mesh] OR "Telemedicine"[Mesh] OR "Monitoring, Physiologic"[Mesh] OR "electronic health record*"[tiab] OR "electronic medical record*"[tiab] OR EHR[tiab] OR EMR[tiab] OR "clinical information system*"[tiab] OR "hospital information system*"[tiab] OR "nursing information system*"[tiab] OR "clinical decision support"[tiab] OR CDSS[tiab] OR "artificial intelligence"[tiab] OR "machine learning"[tiab] OR "internet of things"[tiab] OR IoT[tiab] OR "smart hospital*"[tiab] OR "smart ward*"[tiab] OR "digital transformation"[tiab] OR "digital health"[tiab] OR "ambient clinical documentation"[tiab] OR "ambient documentation"[tiab] OR "speech recognition"[tiab] OR "robotic process automation"[tiab] OR "workflow automation"[tiab] OR "barcode medication administration"[tiab] OR "closed-loop medication"[tiab] OR "remote patient monitoring"[tiab] OR telehealth[tiab]) AND
("Leadership"[Mesh] OR "Professional Competence"[Mesh] OR "Organizational Innovation"[Mesh] OR "Diffusion of Innovation"[Mesh] OR "Implementation Science"[Mesh] OR "nursing leadership"[tiab] OR "nurse manager*"[tiab] OR "nurse leader*"[tiab] OR "digital leadership"[tiab] OR "implementation leadership"[tiab] OR "leadership readiness"[tiab] OR "organizational readiness"[tiab] OR "digital competence"[tiab] OR "digital competency"[tiab] OR "digital literacy"[tiab] OR "AI literacy"[tiab] OR "informatics competence"[tiab] OR "informatics competency"[tiab] OR "change management"[tiab] OR "organizational change"[tiab] OR governance[tiab] OR "clinical governance"[tiab] OR "information governance"[tiab] OR "data governance"[tiab] OR "interoperability governance"[tiab] OR "decision rights"[tiab] OR accountability[tiab] OR "nursing representation"[tiab] OR "nurse participation"[tiab] OR "participatory design"[tiab] OR co-design[tiab] OR "workforce development"[tiab] OR training[tiab] OR education[tiab] OR procurement[tiab] OR "technology evaluation"[tiab] OR "implementation strateg*"[tiab] OR "organizational support"[tiab]) AND
("2015-01-01"[Date - Publication] : "2026-08-21"[Date - Publication])

S2.5 Exact OpenAlex searches

**search_openalex_oa1_20260821**

**Family:** technology_work

hospital nursing electronic health record workflow documentation

**Execution:** Reported results: 11,323. Retrieved: 200. Retrieval ceiling: 200. Status: complete_with_result_ceiling. Search window: 1 January 2015 to 21 August 2026.

**search_openalex_oa2_20260821**

**Family:** technology_work

hospital nurses digital transformation smart hospital nursing informatics

**Execution:** Reported results: 2,415. Retrieved: 200. Retrieval ceiling: 200. Status: complete_with_result_ceiling. Search window: 1 January 2015 to 21 August 2026.

**search_openalex_oa3_20260821**

**Family:** data_burden

nursing documentation burden electronic health record hospital

**Execution:** Reported results: 20,355. Retrieved: 200. Retrieval ceiling: 200. Status: complete_with_result_ceiling. Search window: 1 January 2015 to 21 August 2026.

**search_openalex_oa4_20260821**

**Family:** data_burden

nurses cognitive workload workflow interruption electronic health record

**Execution:** Reported results: 1,577. Retrieved: 200. Retrieval ceiling: 200. Status: complete_with_result_ceiling. Search window: 1 January 2015 to 21 August 2026.

**search_openalex_oa5_20260821**

**Family:** data_burden

nurses clinical decision support alert fatigue hospital

**Execution:** Reported results: 10,938. Retrieved: 200. Retrieval ceiling: 200. Status: complete_with_result_ceiling. Search window: 1 January 2015 to 21 August 2026.

**search_openalex_oa6_20260821**

**Family: data_burden**

nurses physiologic monitor alarm fatigue hospital

**Execution:** Reported results: 3,754. Retrieved: 200. Retrieval ceiling: 200. Status: complete_with_result_ceiling. Search window: 1 January 2015 to 21 August 2026.

**search_openalex_oa7_20260821**

**Family:** data_burden

nursing information system data quality data integrity stewardship

**Execution:** Reported results: 3,481. Retrieved: 200. Retrieval ceiling: 200. Status: complete_with_result_ceiling. Search window: 1 January 2015 to 21 August 2026.

**search_openalex_oa8_20260821**

**Family:** leadership_governance

nurse manager digital leadership hospital technology implementation

**Execution:** Reported results: 15,272. Retrieved: 200. Retrieval ceiling: 200. Status: complete_with_result_ceiling. Search window: 1 January 2015 to 21 August 2026.

**search_openalex_oa9_20260821**

**Family:** leadership_governance

nursing informatics governance hospital organizational readiness

**Execution:** Reported results: 2,085. Retrieved: 200. Retrieval ceiling: 200. Status: complete_with_result_ceiling. Search window: 1 January 2015 to 21 August 2026.

**search_openalex_oa10_20260821**

**Family:** leadership_governance

nurse participation co-design digital health implementation hospital

**Execution:** Reported results: 31,773. Retrieved: 200. Retrieval ceiling: 200. Status: complete_with_result_ceiling. Search window: 1 January 2015 to 21 August 2026.

**search_openalex_oa11_20260821**

**Family:** technology_work

artificial intelligence nursing workflow hospital implementation

**Execution:** Reported results: 10,325. Retrieved: 200. Retrieval ceiling: 200. Status: complete_with_result_ceiling. Search window: 1 January 2015 to 21 August 2026.

**search_openalex_oa12_20260821**

**Family:** context_equity

low resource hospital nursing digital health implementation

**Execution:** Reported results: 45,348. Retrieved: 200. Retrieval ceiling: 200. Status: complete_with_result_ceiling. Search window: 1 January 2015 to 21 August 2026.

**search_openalex_oa13_20260821**

**Family:** context_equity

China hospital nursing information system digital transformation

**Execution:** Reported results: 12,026. Retrieved: 200. Retrieval ceiling: 200. Status: complete_with_result_ceiling. Search window: 1 January 2015 to 21 August 2026.

**search_openalex_oa14_20260821**

**Family:** leadership_governance

hospital nursing digital competence training implementation support

**Execution:** Reported results: 24,271. Retrieved: 200. Retrieval ceiling: 200. Status: complete_with_result_ceiling. Search window: 1 January 2015 to 21 August 2026.
