# Supplementary material for: Hospital Digital Transformation, Nursing Data Burden, and Leadership and Governance Capacity: A Critical Narrative Review
Source: J Nurs Manag. 2026 Oct 6;2026:9681970. doi: 10.1155/jonm/9681970 (PMC13639933; doi:10.1155/jonm/9681970)
Supplement: Supplementary file 3 [file JONM-2026-9681970-s003.docx]

**Supplementary Appendix S3. Claim-to-source map**

This appendix maps 18 conceptual claims to 88 source relationships involving 49 of the 58 cited evidence records. Support is classified as direct, indirect, or contradictory. Every relationship was verified against a full-text location, and source locations identify the section, table, or other location examined during citation-to-claim verification.

| **Claim** | **Claim text** | **Relationship** | **Source** | **Design** | **Access** | **Source location** |
| --- | --- | --- | --- | --- | --- | --- |
| C01 | Hospital digital systems reconfigure nursing work, but effects depend on usability, workflow fit, staffing, and implementation conditions. | direct | Electronic health record system use and documentation burden of acute and critical care nurse clinicians: a mixed-methods study. rec_pmid_39259920 | mixed-methods study | pmc full text | full-text keyword location; evidence_synthesis/fulltext/pmc/PMC11491602.txt |
|  |  | direct | Electronic Health Record Usability: Associations With Nurse and Patient Outcomes in Hospitals. rec_pmid_33797506 | cross-sectional linked-data analysis | pmc full text | DISCUSSION; evidence_synthesis/fulltext/pmc/PMC8187272.sections.json |
|  |  | direct | Impact of Remote Patient Monitoring Systems on Nursing Time, Healthcare Providers, and Patient Satisfaction in General Wards. rec_pmid_38966455 | mixed-methods observational evaluation | pmc full text | Discussion; evidence_synthesis/fulltext/pmc/PMC11223723.sections.json |
|  |  | direct | Use of the Smart Excretion Care System Linked to Electronic Medical Records to Alleviate Nursing Burden and Enhance Patient Convenience: Mixed Methods Study. rec_pmid_37902820 | qualitative focus-group study | pmc full text | Principal Findings; evidence_synthesis/fulltext/pmc/PMC10644194.sections.json |
|  |  | direct | Factors Associated With the Usability and Adoption of Continuous Monitoring Devices With Deterioration Alerting Systems in Acute Hospital Non-ICU Settings: A Mixed Methods Study. rec_pmid_41873534 | convergent mixed-methods study | pmc full text | 1.1. Background; evidence_synthesis/fulltext/pmc/PMC13140944.sections.json |
| C02 | The effect of EHR implementation on nursing time and bedside care is heterogeneous rather than uniformly beneficial or harmful. | direct | Electronic health record system use and documentation burden of acute and critical care nurse clinicians: a mixed-methods study. rec_pmid_39259920 | mixed-methods study | pmc full text | full-text keyword location; evidence_synthesis/fulltext/pmc/PMC11491602.txt |
|  |  | direct | Time-motion examination of electronic health record utilization and clinician workflows indicate frequent task switching and documentation burden. rec_pmid_33936464 | time-motion observational study | pmc full text | full-text keyword location; evidence_synthesis/fulltext/pmc/PMC8075533.txt |
|  |  | contradictory | Impact of Remote Patient Monitoring Systems on Nursing Time, Healthcare Providers, and Patient Satisfaction in General Wards. rec_pmid_38966455 | mixed-methods observational evaluation | pmc full text | Results; evidence_synthesis/fulltext/pmc/PMC11223723.sections.json |
|  |  | contradictory | Use of the Smart Excretion Care System Linked to Electronic Medical Records to Alleviate Nursing Burden and Enhance Patient Convenience: Mixed Methods Study. rec_pmid_37902820 | qualitative focus-group study | pmc full text | Theme 1: Reduced Workload; evidence_synthesis/fulltext/pmc/PMC10644194.sections.json |
|  |  | indirect | Electronic Health Record Adoption and Nurse Reports of Usability and Quality of Care: The Role of Work Environment. rec_pmid_30786302 | secondary cross-sectional analysis | pmc full text | full-text Methods, Results, and Discussion; evidence_synthesis/fulltext/pmc/PMC6382496.sections.json |
| C03 | Nursing data burden includes documentation time, workflow fragmentation, cognitive workload, redundant entry, navigation problems, and perceived burden. | direct | Measurement of clinical documentation burden among physicians and nurses using electronic health records: a scoping review. rec_pmid_33434273 | scoping review | pmc full text | DISCUSSION; evidence_synthesis/fulltext/pmc/PMC8068426.sections.json |
|  |  | direct | Electronic health record system use and documentation burden of acute and critical care nurse clinicians: a mixed-methods study. rec_pmid_39259920 | mixed-methods study | pmc full text | full-text keyword location; evidence_synthesis/fulltext/pmc/PMC11491602.txt |
|  |  | direct | Time-motion examination of electronic health record utilization and clinician workflows indicate frequent task switching and documentation burden. rec_pmid_33936464 | time-motion observational study | pmc full text | full-text keyword location; evidence_synthesis/fulltext/pmc/PMC8075533.txt |
|  |  | direct | Workflow interruption and nurses' mental workload in electronic health record tasks: An observational study. rec_pmid_36890555 | prospective observational study | pmc full text | Background; evidence_synthesis/fulltext/pmc/PMC9996908.sections.json |
|  |  | direct | Quantifying and Visualizing Nursing Flowsheet Documentation Burden in Acute and Critical Care. rec_pmid_30815074 | retrospective descriptive EHR-data analysis | pmc full text | full-text keyword location; evidence_synthesis/fulltext/pmc/PMC6371331.txt |
| C04 | Documentation burden is defined and measured inconsistently, so a universal percentage of shift time cannot be defended from the available evidence. | direct | Measurement of clinical documentation burden among physicians and nurses using electronic health records: a scoping review. rec_pmid_33434273 | scoping review | pmc full text | DISCUSSION; evidence_synthesis/fulltext/pmc/PMC8068426.sections.json |
|  |  | direct | Quantifying and Visualizing Nursing Flowsheet Documentation Burden in Acute and Critical Care. rec_pmid_30815074 | retrospective descriptive EHR-data analysis | pmc full text | full-text keyword location; evidence_synthesis/fulltext/pmc/PMC6371331.txt |
|  |  | direct | Variations in Nursing Documentation Time in a Mental Health Setting: A Retrospective Observational Study of EHR Usage Data. rec_pmid_41349583 | retrospective observational EHR-usage study | pmc full text | full-text keyword location; evidence_synthesis/fulltext/pmc/PMC12680479.txt |
|  |  | indirect | Time-motion examination of electronic health record utilization and clinician workflows indicate frequent task switching and documentation burden. rec_pmid_33936464 | time-motion observational study | pmc full text | full-text keyword location; evidence_synthesis/fulltext/pmc/PMC8075533.txt |
| C05 | Alarm and alert burden can be reduced through customization, rationalization, and unit-level management bundles, although fatigue may persist. | direct | Impact of Alarm Fatigue on the Work of Nurses in an Intensive Care Environment-A Systematic Review. rec_pmid_33202907 | systematic review | pmc full text | 4.1. Theoretical Implications; evidence_synthesis/fulltext/pmc/PMC7697990.sections.json |
|  |  | direct | Testing physiologic monitor alarm customization software to reduce alarm rates and improve nurses' experience of alarms in a medical intensive care unit. rec_pmid_30335824 | pre-post intervention study | pmc full text | Introduction; evidence_synthesis/fulltext/pmc/PMC6193710.sections.json |
|  |  | direct | Development and initial implementation of electronic clinical decision supports for recognition and management of hospital-acquired acute kidney injury. rec_pmid_33148237 | multimethod development and usability evaluation | pmc full text | full-text Methods, Results, and Discussion; evidence_synthesis/fulltext/pmc/PMC7640650.sections.json |
|  |  | direct | A Discount Approach to Reducing Nursing Alert Burden. rec_pmid_38876466 | quality-improvement before-after evaluation | pmc full text | full-text keyword location; evidence_synthesis/fulltext/pmc/PMC11374459.txt |
|  |  | indirect | Human Factors Considerations in Transitions in Care Clinical Decision Support System Implementation Studies. rec_pmid_35308926 | targeted literature review | pmc full text | full-text Methods, Results, and Discussion; evidence_synthesis/fulltext/pmc/PMC8861703.sections.json |
| C06 | Alarm and alert fatigue are important work and safety signals, but the available evidence does not establish that they cause burnout or clinical errors. | direct | Impact of Alarm Fatigue on the Work of Nurses in an Intensive Care Environment-A Systematic Review. rec_pmid_33202907 | systematic review | pmc full text | 1.2. Nursing Staff Overload—Causes and Effects; evidence_synthesis/fulltext/pmc/PMC7697990.sections.json |
|  |  | indirect | Medical and nursing clinician perspectives on the usability of the hospital electronic medical record: A qualitative analysis. rec_pmid_36866778 | qualitative analysis of free-text survey responses | pmc full text | full-text Methods, Results, and Discussion; evidence_synthesis/fulltext/pmc/PMC11401339.sections.json |
|  |  | indirect | Experiences, barriers and expectations regarding current patient monitoring systems among ICU nurses in a University Hospital in Lebanon: a qualitative study. rec_pmid_38440198 | qualitative study | pmc full text | full-text Methods, Results, and Discussion; evidence_synthesis/fulltext/pmc/PMC10910027.sections.json |
|  |  | indirect | Understanding the Role of Clinical Decision Support Systems Among Hospital Nurses Using the FITT (Fit Between Individuals, Tasks, and Technology) Framework: Qualitative Study. rec_pmid_40996085 | qualitative study | pmc full text | full-text Methods, Results, and Discussion; evidence_synthesis/fulltext/pmc/PMC12511814.sections.json |
| C07 | Nursing data-quality risks arise from system design, workflow mismatch, terminology, duplicated fields, and human-machine-environment interactions, not only from individual compliance. | direct | Factors Influencing Information Distortion in Electronic Nursing Records: Qualitative Study. rec_pmid_40202777 | qualitative study | pmc full text | Introduction; evidence_synthesis/fulltext/pmc/PMC12018866.sections.json |
|  |  | direct | Defining an Essential Clinical Dataset for Admission Patient History to Reduce Nursing Documentation Burden. rec_pmid_32643778 | multiorganizational consensus and implementation evaluation | pmc full text | full-text keyword location; evidence_synthesis/fulltext/pmc/PMC7344356.txt |
|  |  | direct | Knowledge-Attitude-Practice Toward Standardized Nursing Terminology Among Nurses in a Tertiary Hospital in Southwest China: A Single-Center Cross-Sectional Survey. rec_pmid_41609141 | single-center cross-sectional survey | open pdf full text | full-text keyword location; evidence_synthesis/fulltext/openalex_oa/rec_pmid_41609141.txt |
|  |  | direct | Influencing factors of self-reported near-miss in clinical nursing information system tasks among nurses: a multicenter cross-sectional study. rec_pmid_40696341 | multicenter cross-sectional study | pmc full text | Discussion; evidence_synthesis/fulltext/pmc/PMC12281732.sections.json |
|  |  | indirect | The effect of nursing participation in the design of a critical care information system: a case study in a Chinese hospital. rec_pmid_29212480 | descriptive case study with pre-post evaluation | pmc full text | Reducing nursing documentation time and allowing more time on bed-side nursing tasks; evidence_synthesis/fulltext/pmc/PMC5719644.sections.json |
| C08 | Digital literacy and informatics competence are associated with readiness, adoption, and clinical decision-making, but the evidence is dominated by cross-sectional self-report studies. | direct | Digital Skills and Readiness of Greek Nurses for Artificial Intelligence Adoption in Clinical Nursing Practice. rec_pmid_42042656 | cross-sectional survey | pmc full text | 1.3. Research Objectives, Hypotheses, and Contribution; evidence_synthesis/fulltext/pmc/PMC13118857.sections.json |
|  |  | direct | Nursing Informatics Competency and Effective Clinical Decision-Making among Nurses in Saudi Arabia. rec_pmid_42140235 | cross-sectional descriptive correlational study | pmc full text | full-text keyword location; evidence_synthesis/fulltext/pmc/PMC13287928.txt |
|  |  | direct | Nursing informatics competency and its associated factors among palliative care nurses: an online survey in mainland China. rec_pmid_38443955 | cross-sectional survey | pmc full text | Discussion; evidence_synthesis/fulltext/pmc/PMC10913251.sections.json |
|  |  | direct | Effects of digital literacy and nursing informatics competency as job resources on nurses’ burnout and work engagement: a cross-sectional study rec_pmid_41430256 | cross-sectional study | open pdf full text | full-text keyword location; evidence_synthesis/fulltext/openalex_oa/rec_pmid_41430256.txt |
|  |  | direct | Nurses' attitudes toward artificial intelligence: AI literacy as a predictor and the mediating effect of AI anxiety. rec_pmid_41272773 | cross-sectional correlational study | pmc full text | Discussion; evidence_synthesis/fulltext/pmc/PMC12752257.sections.json |
| C09 | Individual competence cannot substitute for adequate training, technical support, usable systems, staffing, and workflow-centered implementation. | direct | Electronic Health Record Implementations and Insufficient Training Endanger Nurses' Well-being: Cross-sectional Survey Study. rec_pmid_34941546 | cross-sectional survey | pmc full text | Comparison With Prior Work; evidence_synthesis/fulltext/pmc/PMC8738988.sections.json |
|  |  | direct | Electronic health record system use and documentation burden of acute and critical care nurse clinicians: a mixed-methods study. rec_pmid_39259920 | mixed-methods study | pmc full text | full-text keyword location; evidence_synthesis/fulltext/pmc/PMC11491602.txt |
|  |  | direct | Factors Associated With the Usability and Adoption of Continuous Monitoring Devices With Deterioration Alerting Systems in Acute Hospital Non-ICU Settings: A Mixed Methods Study. rec_pmid_41873534 | convergent mixed-methods study | pmc full text | 4. Discussion; evidence_synthesis/fulltext/pmc/PMC13140944.sections.json |
|  |  | indirect | Nurse Managers' Perspectives on Digital Transformation and Informatics Competencies in E-Leadership: A Qualitative Study. rec_pmid_40726545 | qualitative descriptive study | pmc full text | 3.2.7. Theme VI: Recommendations to Strengthen E-Leadership; evidence_synthesis/fulltext/pmc/PMC12303638.sections.json |
|  |  | indirect | Perspectives and Experiences of Nurse Managers on the Impact of Artificial Intelligence on Nursing Work Environments and Managerial Processes: A Qualitative Study. rec_pmid_40528654 | qualitative descriptive study | pmc full text | Introduction; evidence_synthesis/fulltext/pmc/PMC12174894.sections.json |
| C10 | Nursing leadership shapes implementation climate, workforce preparation, and adaptation to EHR and AI systems, although outcome-based leadership evaluations remain limited. | direct | Nurse Managers' Perspectives on Digital Transformation and Informatics Competencies in E-Leadership: A Qualitative Study. rec_pmid_40726545 | qualitative descriptive study | pmc full text | 1.2. Significance of the Study; evidence_synthesis/fulltext/pmc/PMC12303638.sections.json |
|  |  | direct | Perspectives and Experiences of Nurse Managers on the Impact of Artificial Intelligence on Nursing Work Environments and Managerial Processes: A Qualitative Study. rec_pmid_40528654 | qualitative descriptive study | pmc full text | IMPLICATIONS FOR NURSING PRACTICE AND NURSING POLICY; evidence_synthesis/fulltext/pmc/PMC12174894.sections.json |
|  |  | direct | Nursing Leadership Style, Training Methods, and Use of Electronic Health Records by Nurses in Jordanian Hospitals: A Descriptive Study. rec_pmid_35699627 | cross-sectional survey | pmc full text | Introduction; evidence_synthesis/fulltext/pmc/PMC9449718.sections.json |
|  |  | indirect | The Relationship Between Nursing Managers' Leadership Styles and Nurses' Artificial Intelligence Literacy: A Cross-Sectional Study. rec_pmid_41952446 | cross-sectional study | pmc full text | 4. Discussion; evidence_synthesis/fulltext/pmc/PMC13062651.sections.json |
|  |  | indirect | Artificial intelligence in health care: Assessing impact on professional roles and preparedness among hospital nurse leaders. rec_pmid_40631348 | descriptive correlational cross-sectional study | pmc full text | Results; evidence_synthesis/fulltext/pmc/PMC12235227.sections.json |
| C11 | Governance capacity extends beyond leadership style to nursing representation, co-design, data standards, accountability, and explicit decision processes. | direct | The effect of nursing participation in the design of a critical care information system: a case study in a Chinese hospital. rec_pmid_29212480 | descriptive case study with pre-post evaluation | pmc full text | Methods, tools and techniques for participatory design; evidence_synthesis/fulltext/pmc/PMC5719644.sections.json |
|  |  | direct | Exploratory Co-Design on Electronic Health Record Nursing Summaries: Case Study. rec_pmid_40068168 | exploratory co-design mixed-methods case study | pmc full text | Background; evidence_synthesis/fulltext/pmc/PMC11918977.sections.json |
|  |  | direct | Knowledge-Attitude-Practice Toward Standardized Nursing Terminology Among Nurses in a Tertiary Hospital in Southwest China: A Single-Center Cross-Sectional Survey. rec_pmid_41609141 | single-center cross-sectional survey | open pdf full text | full-text keyword location; evidence_synthesis/fulltext/openalex_oa/rec_pmid_41609141.txt |
|  |  | indirect | Factors Influencing Information Distortion in Electronic Nursing Records: Qualitative Study. rec_pmid_40202777 | qualitative study | pmc full text | Principal Findings; evidence_synthesis/fulltext/pmc/PMC12018866.sections.json |
|  |  | indirect | Nurse Managers' Perspectives on Digital Transformation and Informatics Competencies in E-Leadership: A Qualitative Study. rec_pmid_40726545 | qualitative descriptive study | pmc full text | 1.2. Significance of the Study; evidence_synthesis/fulltext/pmc/PMC12303638.sections.json |
| C12 | Nurse participation and co-design can improve information fit and usability, but supporting studies are mainly case-based, qualitative, or implementation-specific. | direct | The effect of nursing participation in the design of a critical care information system: a case study in a Chinese hospital. rec_pmid_29212480 | descriptive case study with pre-post evaluation | pmc full text | Background; evidence_synthesis/fulltext/pmc/PMC5719644.sections.json |
|  |  | direct | Exploratory Co-Design on Electronic Health Record Nursing Summaries: Case Study. rec_pmid_40068168 | exploratory co-design mixed-methods case study | pmc full text | Background; evidence_synthesis/fulltext/pmc/PMC11918977.sections.json |
|  |  | direct | Evaluation of IMPALA 2.0: Addressing Patient Monitoring in Low-Resource Hospitals in Malawi. rec_pmid_39711002 | mixed-methods usability evaluation | pmc full text | Discussion; evidence_synthesis/fulltext/pmc/PMC11674287.sections.json |
|  |  | direct | Nurses' Adoption, Perceived Usability, and Satisfaction with an Updated Electronic Handover Page Within the Electronic Medical Record: A Mixed-Methods Study. rec_pmid_41149684 | pre-post mixed-methods study | pmc full text | 4. Discussion; evidence_synthesis/fulltext/pmc/PMC12567379.sections.json |
|  |  | indirect | Development and initial implementation of electronic clinical decision supports for recognition and management of hospital-acquired acute kidney injury. rec_pmid_33148237 | multimethod development and usability evaluation | pmc full text | full-text Methods, Results, and Discussion; evidence_synthesis/fulltext/pmc/PMC7640650.sections.json |
| C13 | AI-enabled tools may improve documentation efficiency and usability while introducing hallucination, trust-calibration, oversight, and new alert-burden concerns. | direct | Integrating a Large Language Model to Streamline Nursing Handover Documentation Across Multiple Hospitals in Taiwan: Development and Implementation Study. rec_pmid_41819121 | multihospital before-after implementation study | pmc full text | Safety Considerations and Hallucination Mitigation; evidence_synthesis/fulltext/pmc/PMC13022550.sections.json |
|  |  | direct | Implementation of an AI-Assisted Workflow for Rehabilitation Discharge Summary Creation: Evaluation of Documentation Efficiency, Usability, and Hallucinations in a 290-Bed General Hospital in Japan. rec_pmid_42326256 | single-center before-after implementation study | pmc full text | Discussion; evidence_synthesis/fulltext/pmc/PMC13282032.sections.json |
|  |  | direct | Spatially Integrated Electronic Health Record Prototype Improves Usability and Reduces Cognitive Load for Intensive Care Nurses: A Randomized Crossover Trial. rec_pmid_42486461 | randomized crossover trial | pmc full text | Methods, Results, and Discussion; evidence_synthesis/fulltext/pmc/PMC13391218.sections.json |
|  |  | direct | Why Nurses Intend to Override AI Alerts: How Alert Fatigue, Moral Distress, and Team Psychological Safety Shape Self-Reported Trust Calibration Toward Clinical Decision Support. rec_pmid_42512579 | cross-sectional survey | pmc full text | 3. Results; evidence_synthesis/fulltext/pmc/PMC13410929.sections.json |
|  |  | indirect | Perspectives and Experiences of Nurse Managers on the Impact of Artificial Intelligence on Nursing Work Environments and Managerial Processes: A Qualitative Study. rec_pmid_40528654 | qualitative descriptive study | pmc full text | Introduction; evidence_synthesis/fulltext/pmc/PMC12174894.sections.json |
| C14 | Resource constraints, infrastructure reliability, interoperability, and local workflow conditions modify the feasibility and transferability of hospital digital interventions. | direct | Evaluation of IMPALA 2.0: Addressing Patient Monitoring in Low-Resource Hospitals in Malawi. rec_pmid_39711002 | mixed-methods usability evaluation | pmc full text | Discussion; evidence_synthesis/fulltext/pmc/PMC11674287.sections.json |
|  |  | direct | Comprehensive evaluation of electronic medical record system use and user satisfaction at five low-resource setting hospitals in ethiopia. rec_pmid_26007237 | cross-sectional evaluation study | pmc full text | System Detail and System in Use; evidence_synthesis/fulltext/pmc/PMC4460264.sections.json |
|  |  | indirect | A cross-sectional analysis of AI readiness and attitudes among nurses in resource-limited Chinese county hospitals. rec_pmid_41867482 | cross-sectional study | pmc full text | Introduction; evidence_synthesis/fulltext/pmc/PMC12999563.sections.json |
|  |  | indirect | Acceptability and usability of a nurse-assisted remote patient monitoring intervention for the post-hospital follow-up of patients with long-term illness: A qualitative study. rec_pmid_39166216 | qualitative study | pmc full text | full-text Methods, Results, and Discussion; evidence_synthesis/fulltext/pmc/PMC11334779.sections.json |
| C15 | Evidence from China illustrates rapid experimentation with nursing information systems, AI readiness, and digital leadership, but much of the recent literature is local and cross-sectional. | direct | The effect of nursing participation in the design of a critical care information system: a case study in a Chinese hospital. rec_pmid_29212480 | descriptive case study with pre-post evaluation | pmc full text | Background; evidence_synthesis/fulltext/pmc/PMC5719644.sections.json |
|  |  | direct | The impact of a 5G-based smart nursing information system and associated mobile hardware on clinical nurses' work stress: a randomized controlled study in a Chinese hospital. rec_pmid_39923066 | randomized controlled trial | pmc full text | Background; evidence_synthesis/fulltext/pmc/PMC11806821.sections.json |
|  |  | direct | Factors Influencing Information Distortion in Electronic Nursing Records: Qualitative Study. rec_pmid_40202777 | qualitative study | pmc full text | Principal Findings; evidence_synthesis/fulltext/pmc/PMC12018866.sections.json |
|  |  | direct | Knowledge-Attitude-Practice Toward Standardized Nursing Terminology Among Nurses in a Tertiary Hospital in Southwest China: A Single-Center Cross-Sectional Survey. rec_pmid_41609141 | single-center cross-sectional survey | open pdf full text | full-text keyword location; evidence_synthesis/fulltext/openalex_oa/rec_pmid_41609141.txt |
|  |  | direct | Technophobia and Its Associated Factors Among Registered Nurses in China: A Cross-Sectional Multicenter Study. rec_pmid_41992642 | multicenter cross-sectional study | pmc full text | 4. Discussions; evidence_synthesis/fulltext/pmc/PMC13087445.sections.json |
|  |  | direct | Artificial intelligence literacy and influencing factors among clinical nurses in southeastern China: a latent profile analysis. rec_pmid_42271407 | cross-sectional latent-profile analysis | pmc full text | Current status and heterogeneity of AI literacy; evidence_synthesis/fulltext/pmc/PMC13474754.sections.json |
|  |  | direct | A cross-sectional analysis of AI readiness and attitudes among nurses in resource-limited Chinese county hospitals. rec_pmid_41867482 | cross-sectional study | pmc full text | Introduction; evidence_synthesis/fulltext/pmc/PMC12999563.sections.json |
| C16 | The technology-work, data-burden, and leadership-governance triad is an evidence-informed organizing heuristic, not a validated causal or mediation model. | indirect | Electronic health record system use and documentation burden of acute and critical care nurse clinicians: a mixed-methods study. rec_pmid_39259920 | mixed-methods study | pmc full text | full-text keyword location; evidence_synthesis/fulltext/pmc/PMC11491602.txt |
|  |  | indirect | Measurement of clinical documentation burden among physicians and nurses using electronic health records: a scoping review. rec_pmid_33434273 | scoping review | pmc full text | DISCUSSION; evidence_synthesis/fulltext/pmc/PMC8068426.sections.json |
|  |  | indirect | Nurse Managers' Perspectives on Digital Transformation and Informatics Competencies in E-Leadership: A Qualitative Study. rec_pmid_40726545 | qualitative descriptive study | pmc full text | 1.2. Significance of the Study; evidence_synthesis/fulltext/pmc/PMC12303638.sections.json |
|  |  | indirect | The effect of nursing participation in the design of a critical care information system: a case study in a Chinese hospital. rec_pmid_29212480 | descriptive case study with pre-post evaluation | pmc full text | Discussion; evidence_synthesis/fulltext/pmc/PMC5719644.sections.json |
|  |  | indirect | Evaluation of IMPALA 2.0: Addressing Patient Monitoring in Low-Resource Hospitals in Malawi. rec_pmid_39711002 | mixed-methods usability evaluation | pmc full text | Conclusions; evidence_synthesis/fulltext/pmc/PMC11674287.sections.json |
| C17 | Training and post-implementation support are continuing implementation conditions rather than one-time deployment tasks. | direct | Electronic Health Record Implementations and Insufficient Training Endanger Nurses' Well-being: Cross-sectional Survey Study. rec_pmid_34941546 | cross-sectional survey | pmc full text | Comparison With Prior Work; evidence_synthesis/fulltext/pmc/PMC8738988.sections.json |
|  |  | direct | Testing physiologic monitor alarm customization software to reduce alarm rates and improve nurses' experience of alarms in a medical intensive care unit. rec_pmid_30335824 | pre-post intervention study | pmc full text | Number and duration of alarms; evidence_synthesis/fulltext/pmc/PMC6193710.sections.json |
|  |  | direct | Nursing Leadership Style, Training Methods, and Use of Electronic Health Records by Nurses in Jordanian Hospitals: A Descriptive Study. rec_pmid_35699627 | cross-sectional survey | pmc full text | Introduction; evidence_synthesis/fulltext/pmc/PMC9449718.sections.json |
|  |  | direct | Implementation and delivery of electronic health records training programs for nurses working in inpatient settings: a scoping review. rec_pmid_39225789 | scoping review | pmc full text | full-text keyword location; evidence_synthesis/fulltext/pmc/PMC11491623.txt |
|  |  | indirect | Digital Skills and Readiness of Greek Nurses for Artificial Intelligence Adoption in Clinical Nursing Practice. rec_pmid_42042656 | cross-sectional survey | pmc full text | 5. Conclusions; evidence_synthesis/fulltext/pmc/PMC13118857.sections.json |
| C18 | Associations of digital-system burden with bedside presence and nurse well-being are plausible but variable, so universal burden percentages and deterministic burnout claims should be avoided. | direct | Electronic Health Record Usability: Associations With Nurse and Patient Outcomes in Hospitals. rec_pmid_33797506 | cross-sectional linked-data analysis | pmc full text | DISCUSSION; evidence_synthesis/fulltext/pmc/PMC8187272.sections.json |
|  |  | direct | Electronic Health Record Implementations and Insufficient Training Endanger Nurses' Well-being: Cross-sectional Survey Study. rec_pmid_34941546 | cross-sectional survey | pmc full text | Introduction; evidence_synthesis/fulltext/pmc/PMC8738988.sections.json |
|  |  | direct | Electronic health record system use and documentation burden of acute and critical care nurse clinicians: a mixed-methods study. rec_pmid_39259920 | mixed-methods study | pmc full text | full-text keyword location; evidence_synthesis/fulltext/pmc/PMC11491602.txt |
|  |  | contradictory | Impact of Remote Patient Monitoring Systems on Nursing Time, Healthcare Providers, and Patient Satisfaction in General Wards. rec_pmid_38966455 | mixed-methods observational evaluation | pmc full text | Results; evidence_synthesis/fulltext/pmc/PMC11223723.sections.json |
